# Model of fibrolamellar hepatocellular carcinomas reveals striking enrichment in cancer stem cells

**DOI:** 10.1038/ncomms9070

**Published:** 2015-10-06

**Authors:** Tsunekazu Oikawa, Eliane Wauthier, Timothy A. Dinh, Sara R. Selitsky, Andrea Reyna-Neyra, Guido Carpino, Ronald Levine, Vincenzo Cardinale, David Klimstra, Eugenio Gaudio, Domenico Alvaro, Nancy Carrasco, Praveen Sethupathy, Lola M. Reid

**Affiliations:** 1 Department of Cell Biology and Physiology, Chapel Hill, North Carolina 27599, USA; 2 Program in Molecular Biology and Biotechnology, Chapel Hill, North Carolina 27599, USA; 3 Lineberger Comprehensive Cancer Center, Chapel Hill, North Carolina 27599, USA; 4 Department of Genetics, Chapel Hill, North Carolina 27599, USA; 5 Curriculum in Genetics and Molecular Biology, Chapel Hill, North Carolina 27599, USA; 6 MD-PhD Program, UNC School of Medicine, Chapel Hill, North Carolina 27599, USA; 7 Curriculum in Bioinformatics and Computational Biology, UNC School of Medicine, Chapel Hill, North Carolina 27599, USA; 8 Department of Cellular and Molecular Physiology, Yale University School of Medicine, 333 Cedar Street, New Haven, Connecticut 06510, USA; 9 Department of Movement, Human and Health Sciences, Division of Health Sciences, University of Rome ‘Foro Italico', Piazza Lauro De Bosis 6, 00151 Rome, Italy; 10 Greenwich Hospital, 5 Perryridge Road, Greenwich, Connecticut 06830, USA; 11 Yale University School of Medicine, 333 Cedar Street, New Haven, Connecticut, 06510 USA; 12 Division of Gastroenterology, Department of Scienze e Biotecnologie Medico-Chirurgiche, Fondazione Eleonora Lorillard Spencer Cenci, Polo Pontino, Viale dell'Universita 37, 00185 Rome, Italy; 13 Department of Pathology, Memorial Sloan Kettering Cancer Center, 1275 York Avenue, New York, New York 10065, USA; 14 Department of Anatomical, Histological, Forensic Medicine and Orthopedic Sciences, Sapienza University of Rome, Via Borelli 50, 00161 Rome, Italy

## Abstract

The aetiology of human fibrolamellar hepatocellular carcinomas (hFL-HCCs), cancers occurring increasingly in children to young adults, is poorly understood. We present a transplantable tumour line, maintained in immune-compromised mice, and validate it as a *bona fide* model of hFL-HCCs by multiple methods. RNA-seq analysis confirms the presence of a fusion transcript *(DNAJB1-PRKACA)* characteristic of hFL-HCC tumours. The hFL-HCC tumour line is highly enriched for cancer stem cells as indicated by limited dilution tumourigenicity assays, spheroid formation and flow cytometry. Immunohistochemistry on the hFL-HCC model, with parallel studies on 27 primary hFL-HCC tumours, provides robust evidence for expression of endodermal stem cell traits. Transcriptomic analyses of the tumour line and of multiple, normal hepatic lineage stages reveal a gene signature for hFL-HCCs closely resembling that of biliary tree stem cells—newly discovered precursors for liver and pancreas. This model offers unprecedented opportunities to investigate mechanisms underlying hFL-HCCs pathogenesis and potential therapies.

## Abstract (web summary)

With no cell lines available, investigating the aetiology of human fibrolamellar hepatocellular carcinomas (hFL-HCCs) has proved problematic. Here, Oikawa *et al*. establish a model of hFL-HCCs as a transplantable tumour line maintained in immune-compromised mice, which proves rich in cancer stem cells.

## Graphical abstract

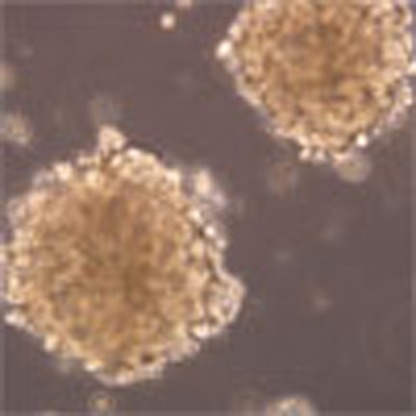

Human fibrolamellar hepatocellular carcinomas (hFL-HCCs) are unique in that they occur primarily in children to young adults without evidence of fibrosis or cirrhosis^1^,^2^,^3^,^4^,^5^. The epidemiological factors are unknown, as are causes of increases in occurrence in hFL-HCCs over the past ∼60 years^6^. These malignances are treatable only by surgery and only if diagnosed before the occurrence of metastases. All forms of chemo and external radiation therapy have proven ineffective. Molecular mechanisms and screens for novel therapies have been difficult to study, since only fresh tissue or paraffin sections have been available, and those are in limited supply. There are no cell lines, and until our studies, no transplantable tumour lines of hFL-HCCs.

We established the first-ever hFL-HCC transplantable tumour line in immune-compromised murine hosts and compared its phenotypic features with those of 27 primary hFL-HCC tumours. The hFL-HCC tumour line proved rich in cancer stem cells (CSCs). The hFL-HCCs were found to be most closely related to normal human biliary tree stem cells (hBTSCs), newly discovered stem cell subpopulations found throughout the biliary tree and now shown to be precursors to both liver and pancreas^7^,^8^,^9^,^10^,^11^,^12^,^13^,^14^.

## Results

### Establishment of a patient-derived xenograft hFL-HCC model

A young male patient was diagnosed with hFL-HCC and was subjected to liver surgery and chemotherapies, all proving unsuccessful. A more detailed presentation of the diagnosis of the tumour and its progression is given in the Supplementary Note 1 and Supplementary Table 1. Within 2 years, the tumour had metastasized and generated ascites tumour cells. Approximately 5 liters of ascites fluid were removed from the patient. Cells from 4 of the liters were delivered to the Reid lab at University of North Carolina (UNC) and were cultured in Kubota's Medium (KM), a serum-free medium found effective for culture selection of endodermal stem/progenitors^7^,^11^,^15^,^16^. Culture-selected cells (2 × 10^7^ cells) were transplanted into NOD SCID gamma (NSG) immune-compromised mice. The initial tumour formation in the mice required >6 months (Table 1).

### Transplantation of the tumour line stabilized by supplements

**Figure 1:**
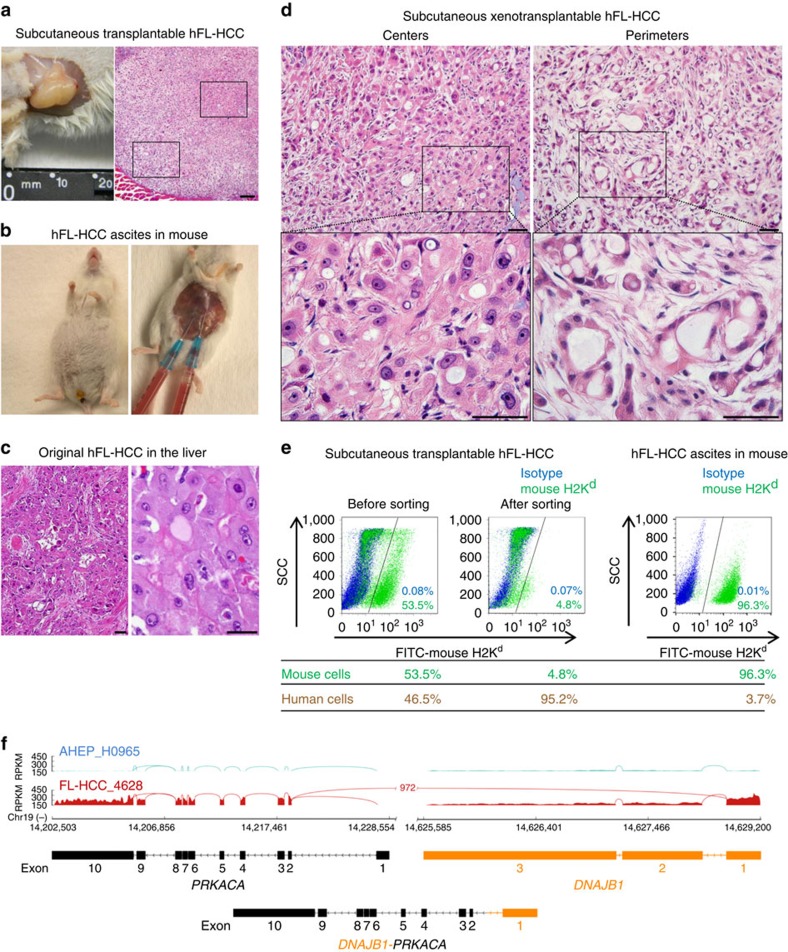
Establishment of a transplantable hFL-HCC tumour line. (**a**) Subcutaneous tumour generated in NSG mice. (**b**) Intraperitoneal transplants resulting in ascites tumours and tumours on serosal surfaces of abdominal organs. (**c**) Hematoxylin/eosin stained sections of the original solid, intrahepatic hFL-HCC tumour. (**d**) Histology of the centres (1) versus perimeters (2) of the subcutaneous xenografted tumour at low and high magnifications. The scale bar, 25 μm (**a**,**c**,**d**). (**e**) Flow cytometric characterizations of tumour cell suspensions from subcutaneous xenografted tumours indicated that over 50% of the cells are host (murine) cells as indicated by expression of H2K^d^ (in data shown, it is 53.5%). Host mesenchymal cells can be minimized to <5% by negative sorting for H2K^d^+ cells. The extent of host mesenchymal cells is greater when tumours are transplanted intraperitoneally; host cells comprised >95% of the cells (data shown indicates 96.3% were host cells). (**f**) Sashimi plot of RNA-seq read coverage for fusion gene *DNAJB1-PRKACA*. The *DNAJB1-PRKACA* chimera was detected only in the cells of the hFL-HCC transplantable tumour line and not in normal hAHEPs. Solid peaks depict reads per kilobase per million reads mapped. Splice/fusion junctions are shown as arcs. The fusion junction joins exon1 of *DNAJB1* with the start of exon2 of *PRKACA*. This fusion gene has been reported previously in FL-HCCs^18^,^19^,^20^.

Tumours were transplanted every 3–5 months. Successful serial transplantation (from mouse to mouse) was stabilized at ∼3 months/passage with transplantation of ∼10^6^ cells in KM supplemented with 1 mg ml^−1^ hyaluronans and with 50 ng ml^−1^ each of hepatocyte growth factor (HGF) and vascular endothelial cell growth factor (VEGF; Fig. 1a and Table 1). The transplantable, subcutaneous tumours were aggressive in being able to penetrate through the body wall into the peritoneum. They were nodular and difficult to mince. If transplanted intraperitoneally (Fig. 1b), ascites tumours formed after ∼8 weeks and gave rise to nodules on all serosal surfaces within the abdomen.

### Histology of the tumour line matched that of original tumour

The histology of the original tumour (Fig. 1c) and of the original ascites tumour cells (Supplementary Fig. 1) versus that of xenografts (Fig. 1d) revealed distinctions between tumour centres and perimeters, sites at which tumours interfaced with host tissues. Tumour centres demonstrated histology similar to that of the original tumour with large polygonal cells, abundant eosinophilic cytoplasm, large, vesiculated nuclei and large nucleoli. By contrast, histology at tumour perimeters comprised incomplete ductular structures with partially stabilized lumens and with features similar to that of intrahepatic, mixed-type cholangiocarcinomas (CCAs) with ductular areas^17^.

### Host mesenchymal cells depleted from xenografts by sorting

Mesenchymal cells within xenografted tumours comprised ∼55–70% of cell suspensions from subcutaneous tumours and >95% of those from intraperitoneal tumours. Enrichment of hFL-HCCs to ⩾95% was achieved by negative sorting using magnetic bead immune selection to eliminate murine cells (positive for H-2 K^d^; Fig. 1e). Tumours contained, on average, ∼8 × 10^6^ hFL-HCC cells g^−1^ of tumour.

### RNA-seq identifies DNAJB1-PRKACA chimera in the tumor line

A signature feature of most primary hFL-HCCs is the expression of a fusion transcript, *DNAJB1-PRKACA*^18^,^19^, which was shown recently in a study of 78 primary tumours to be present in at least ∼80% of FL-HCCs^20^. To determine whether the tumour line has this marker, we performed transcriptomic analysis. Specifically, we carried out paired-end high-throughput RNA sequencing in four different hFL-HCC passages of the transplantable tumour line, as well as in purified populations of human adult hepatocytes (hAHEPs) from three different donors. We obtained an average of ∼150 million paired-end reads per sample, of which an average of ∼83% mapped uniquely to the human genome (Supplementary Table 2). We analysed the sequencing data using MapSplice2 and detected *DNAJB1-PRKACA* with high confidence in all four tumour samples of the hFL-HCC tumour line, but not in any of the hAHEPs (Fig. 1f). These results support interpretation of the transplantable tumour line as a *bona fide* model of hFL-HCC.

### Tumourigenicity assays indicate hFL-HCC is rich in CSCs

Cell suspensions, depleted of murine cells, were transplanted subcutaneously into NSG mice in limited dilution tumourigenicity assays in cell numbers from 100 to 10^6^ cells. The mice were monitored for up to 9 months. Tumours formed within ∼3 months in all mice transplanted with 10^5^ or more cells; within 5–6 months if transplanted with 10^3^–10^4^ cells; and, surprisingly, just 100 cells gave rise to tumours in all mice within 9 months (Table 1). Thus, the hFL-HCC tumour line proved functionally rich in CSCs, albeit slow growing. This caused us to investigate further the expression of stem/progenitor cell markers in the tumour line.

### Stem/progenitor traits detected in the tumour line by IHC

**Figure 2:**
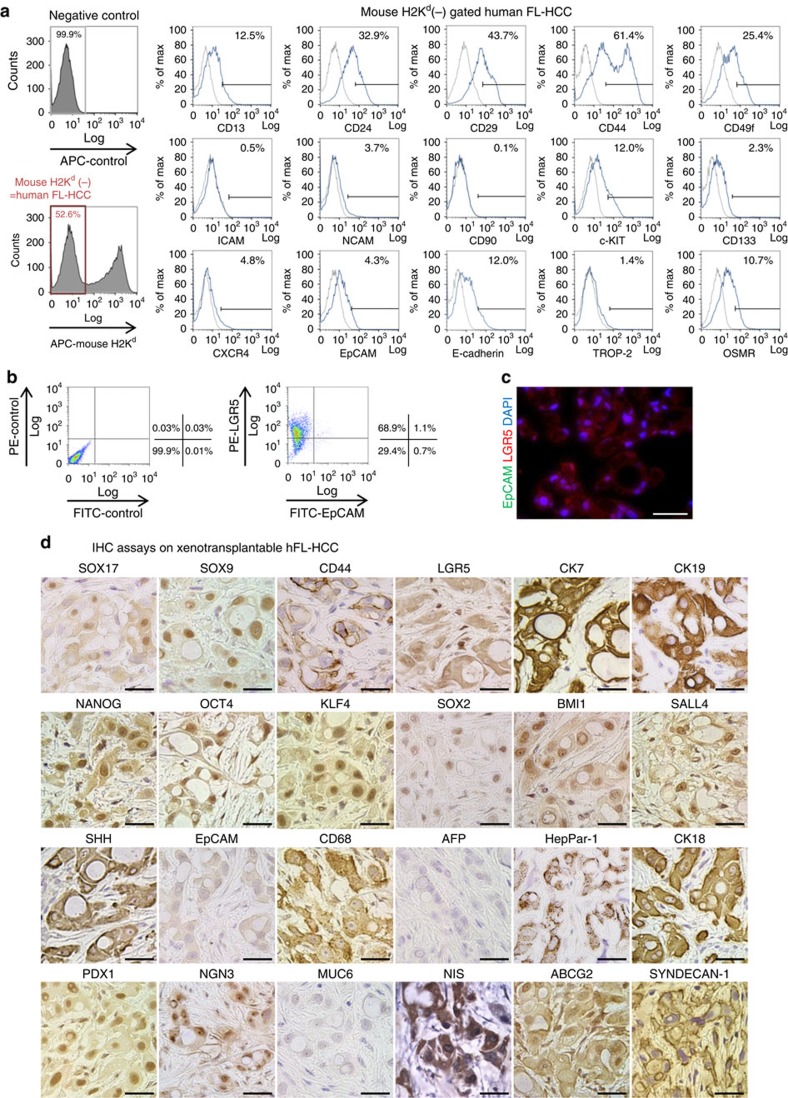
Characterization of a transplantable hFL-HCC tumour line. (**a**) Representative flow cytometric findings of hFL-HCC cells, gated-mouse-H2K^d^-negative, were done. Antigens expressed by a significant percentage of the cells included CD44 (61.4%); CD49f (25.4%); CD24 (32.9%); CD13 (12.5%); c-KIT (12.0%); E-cadherin (12.0%); and OSMR (10.7%). Other antigens found routinely but in a smaller percentage of cells included CXCR4 (4.8%); EpCAM (4.3%); CD133 (2.3%); TROP-2 (1.4%); and ICAM1 (0.5%). (**b**) Double staining of LGR5 and EpCAM in hFL-HCC cells. LGR5+ cells accounted for 68.9% of the cells in the tumours. Of these, only 1.1% were also EpCAM+. (**c**) Immunofluorescence assay on hFL-HCC xenotransplantable tumour demonstrated strong expression of LGR5 and an absence of EpCAM. (**d**) IHC assays on the xenotransplantable tumour line. The survey included assays for endodermal stem/progenitor transcription factors and markers (SOX17, SOX9, CD44, LGR5 and CK19); pluripotency genes (NANOG, OCT4, KLF4, SOX2 and BMI1); hepatic and other markers (HepPar-1, CK18, CK7, SHH and CD68); and pancreatic/endocrine markers (PDX1, NGN3 and NIS), which were strongly expressed. EpCAM was essentially negative, and alpha-fetoprotein was negative. The scale bar, 25 μm (**c**,**d**).

The hFL-HCC cells, flow cytometrically gated away from murine cells, were characterized by multiparametric flow cytometry (Fig. 2a,b). The majority of cells were positive for LGR5 (68.9%) and CD44 (61.4%); a substantial percentage were positive for CD29 (43.7%), CD24 (32.9%), CD49f (25.4%), CD13 (12.5%), E-cadherin (12.0%), c-KIT (12.0%) and oncostatin M receptor-OSMR (10.7%). A low but reproducible percentage of cells were positive for NCAM (3.7%), EpCAM (4.3%), CXCR4 (4.8%), CD133 (2.3%), TROP-2 (1.4%) and ICAM1 (0.5%). A small percentage (1.1%) of LGR5+ cells were positive for EpCAM.

Sections of xenografted hFL-HCC tumours were subjected to Immunohistochemistry (IHC) assays (Fig. 2c,d, and Supplementary Fig. 2a), and the findings were comparable to those from the original tumour cells. The xenografts were positive for CD68, a known trait of hFL-HCCs^21^; stem/progenitor markers (SOX17, SOX9, LGR5, sonic hedgehog (SHH), NCAM and BMI1); pluripotency genes (NANOG, OCT4, KLF4, SOX2 and SALL4); some hepatic markers (HepPar-1, CK7, CK19 and CK18); endocrine pancreatic markers (PDX1); and sodium iodide symporter (NIS). The xenografted tissue was negative or only weakly positive for EpCAM, and negative for alpha-fetoprotein and MUC6. Collectively these traits implicate a very primitive and highly aggressive tumour.

Other markers included matrix components that facilitate cell survival and growth: E-cadherin; syndecan-1 (HS-PG1); hyaluronan receptors (CD44); and vascular cell adhesion molecule-1 (VCAM-1). The cells also strongly expressed multiple genes for multidrug resistance suggestive of insensitivity to diverse chemotherapeutic agents. The cells did not express hemopoietic (CD34 and CD45), stellate (CD146) or endothelial cell antigens (CD31). Negative controls are shown in Supplementary Fig. 2a.

### Stem/progenitor traits also detected in primary hFL-HCCs

**Figure 3:**
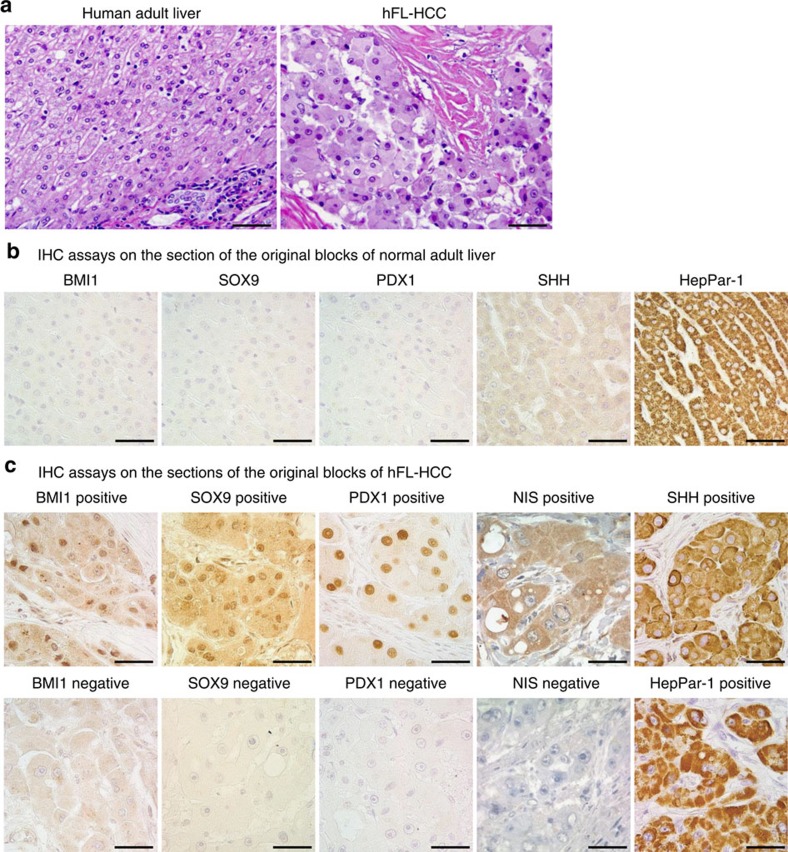
IHC assays on clinical samples of primary tumours of hFL-HCCs compared with normal adult livers. (**a**) Hematoxylin/eosin stained paraffin sections. (**b**,**c**) Representative IHC assays on sections from original blocks of normal adult liver versus primary hFL-HCC tumours. The scale bar, 50 μm (**a**), 25 μm (**b**,**c**).

To determine if the stem cell traits of the transplantable tumour are unique to the tumour line or are evident in primary hFL-HCCs, we obtained paraffin sections from nine original blocks from the Department of Pathology at the Memorial Sloan Kettering Cancer Center (MSKCC) and performed IHC assays (Fig. 3a–c, Table 2 and Supplementary Table 3). All sections assayed were positive for HepPar-1 and SHH. Positive expression was also observed in 7/9 for SOX9, PDX1 and NIS and 4/9 for BMI1.

Tissue microarrays (TMAs) from 18 primary tumours versus 19 normal livers (Table 2 and Supplementary Fig. 2b–c) were also obtained from MSKCC and subjected to IHC. Whereas stem/progenitor traits were not observed in any of the 19 normal livers, all 18 hFL-HCCs were positive for multiple stem/progenitor cell markers: SOX9 (12/18), SOX17 (8/18), PDX1 (13/18), OCT4 (7/18), SALL4 (3/6) and SHH (18/18). Thus, the transplantable hFL-HCC tumour line and the 27 hFL-HCC primary tumours (9 paraffin sections and 18 TMAs) expressed a wealth of markers for endodermal stem cell traits and some pluripotency genes.

### Spheroids, indicative of CSCs, formed in serum-free medium

**Figure 4:**
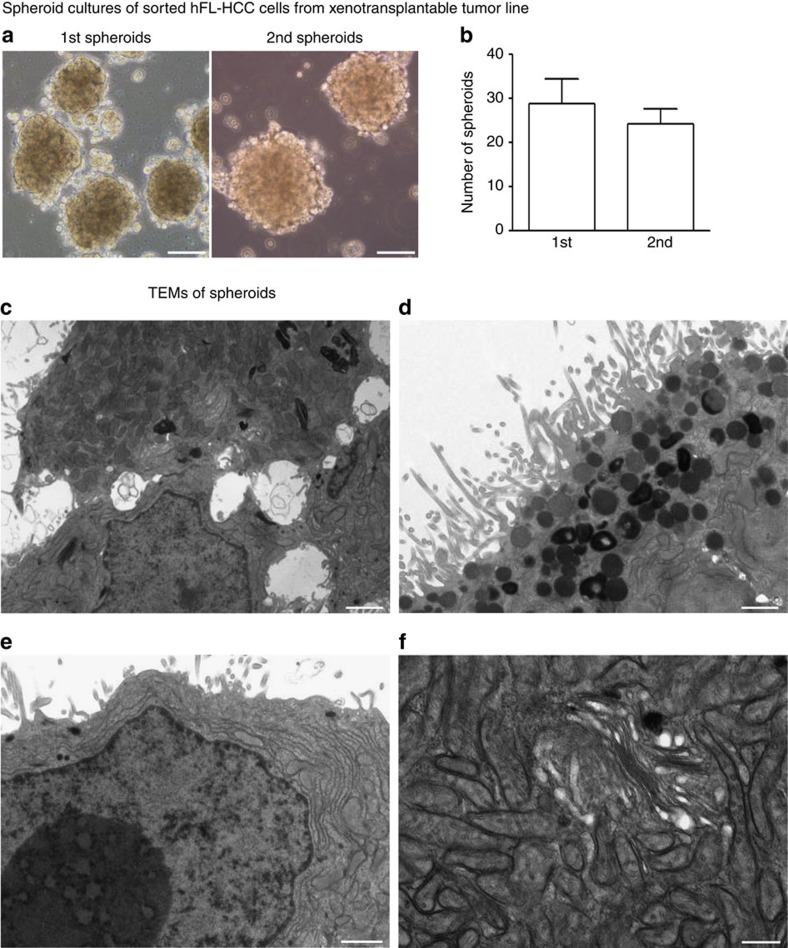
Spheroid cultures of hFL-HCC cells from transplantable tumour line. Plating and maintaining hFL-HCC tumour cells under serum-free conditions, and especially after depletion of the murine cells, resulted in spheroid formation. (**a**) Spheroids formed from freshly isolated hFL-HCC cells depleted of murine cells (1st or primary spheroids) were sustainable in culture for months and were passagable forming secondary spheroids (2nd spheroids). (**b**) The number of spheroids formed from 10,000 seeded cells remained similar in primary and secondary spheroids. Data are represented as the mean spheroids (>100 μm) count±s.d. (triplicate samples). (**c**–**f**) TEM of spheroids. (**c**); tumour cells displayed numerous microvilli at their apical pole and tight junctions, meaning the tumour cells could still polarize. At their apical pole, tumour cells had numerous secretory vesicles containing electron-dense granules. Note the presence of microvilli; large numbers of secretory vesicles with electron-dense granules; partially formed ducts (**d**); nuclei presented dispersed chromatin and large nucleoli implicating production of secretory proteins (**e**); a wealth of mitochondria with aberrant cristae (**f**). The scale bar, 1 μm (**c**–**e**), 200 nm (**f**).

Spheroid formation required that serum-free KM be used throughout, including for plating of cells, and was facilitated further by depletion of host mesenchymal cells from the tumour cell suspensions. The spheroids formed readily, within hours; proved able to be passaged for months; and were able to generate secondary spheroids (Fig. 4a,b). The number of primary and secondary spheroids was significantly increased when they were cultured with 1 mg ml^−1^ hyaluronans (Supplementary Fig. 3a). Transmission electron microscopy (TEM) of spheroids (Fig. 4c–f and Supplementary Fig. 3b–f) revealed tumour cells with microvilli at their apical poles, indicating ability to polarize. Cells were rich in rough endoplasmic reticulum and Golgi, with numerous secretory vesicles containing electron-dense granules typically associated with neuroendocrine traits (for example, chromogranin) common to pancreatic tumours. Nuclei contained dispersed chromatin and large nucleoli, implicating high production of secretory proteins. Cells were rich in pleomorphic, irregularly-shaped, non-lucent mitochondria with irregular disorganized cristae.

### Stem/progenitor traits detected in cultures of hFL-HCC cells

**Figure 5:**
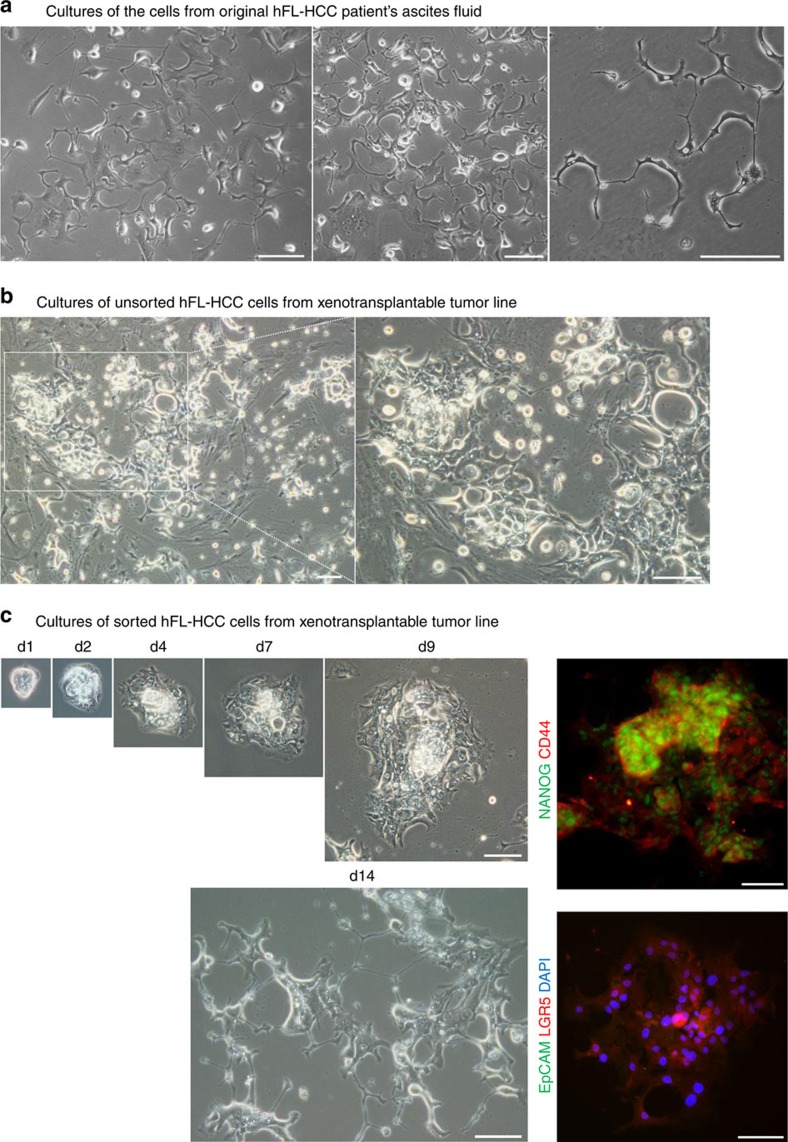
Monolayer cultures of hFL-HCC cells from original ascites fluid and from transplantable tumour line. (**a**) Suspensions of the original hFL-HCC cells from patient's ascites fluid were plated onto culture plastic and in serum-free KM. The cells transiently attached and formed star-like cells. Subsequently, the cells became loosely attached to the dish but remained attached to each other via E-cadherin linkages such that they formed floating cell chains (catena). Cells were readily lost with media changes (**b**) Primary cultures of the transplantable tumour line yielded cells with a similar appearance to the original ascites cells. (**c**) Depletion of the host (murine) cells enabled hFL-HCC cells to form colonies at single cell seeding densities and that grew into colonies within 2 weeks. After 2 weeks, these cells morphologically resembled the cultures of the original ascites cells. The flattened, monolayer cells expressed pluripotency and stem cell genes (NANOG, CD44 and LGR5) but not EpCAM. The scale bar, 100 μm.

The original hFL-HCC cells from ascites fluid attached and formed star-like cells (Fig. 5a) but transitioned rapidly with a day or two into cells loosely attached and connected to floating cell chains (catena), with cells bound to each other via E-cadherin linkages. If plated overnight with 2–5% foetal bovine serum (FBS) and then switched to serum-free KM (Fig. 5b), primary cultures of hFL-HCC cells and associated mesenchymal cells attached to the dishes. Whereas the mesenchymal cells attached and spread, the colonies of hFL-HCCs had some cells that attached initially but then transitioned steadily within a week into aggregates that floated into the medium as spheroids. If depleted of murine cells (Fig. 5c) and plated in serum-free KM, hFL-HCC colonies remained attached longer (shown are cultures up to 2 weeks) expanding to form somewhat three-dimensional (3D) cells with undulating morphologies. These hFL-HCC cells stained positive for NANOG, CD44 and LGR5, but not for EpCAM (Fig. 5c).

### Stem/progenitor traits lost in differentiation media

**Figure 6:**
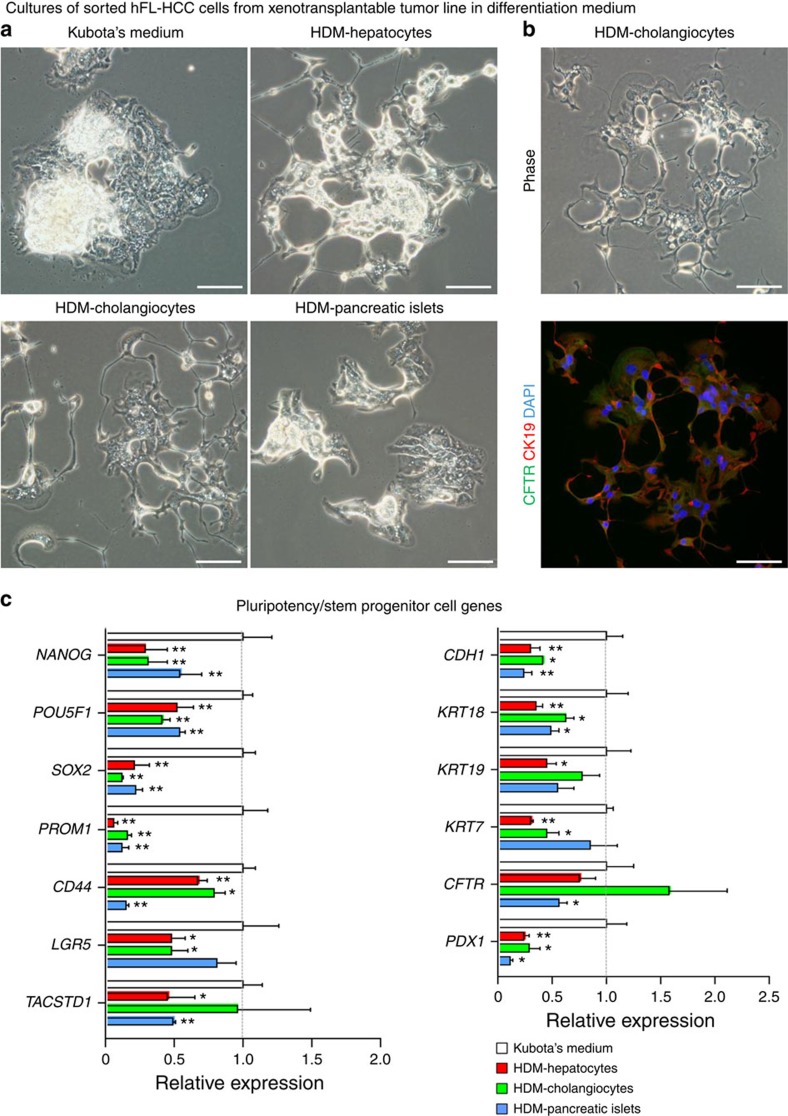
Differentiation assay of FL-HCC cells from transplantable tumour line. (**a**) Expansion versus differentiation conditions for hFL-HCC cells resulted in distinct morphologies of the hFL-HCC colonies and changes in their stem cell versus tissue-specific gene expression. Cultures in serum-free KM retained stemness traits throughout the weeks (to months) of culture. By contrast, cultures in serum-free, HDM tailored for lineage restriction of stem cells to hepatocytes (HDM-H), cholangiocytes (HDM-C) or pancreatic islets (HDM-P) caused transient distinctions in morphology that transitioned secondarily towards spheroid formation. (**b**) During the few days when morphological changes were observed, there was an increase in expression of CFTR, a trait of maturing or mature cholangiocytes. (**c**) qRT–PCR assays showed that stem cell traits (*NANOG, POU5F1, SOX2, PROM1*) were suppressed in all the HDM. Surprisingly, so were *KRT18* and *PDX1* and to a lesser extent *KRT7*. *CD44* was partially suppressed in HDM-H and HDM-C but strongly suppressed by HDM-P; *LGR5* was suppressed in HDM-H and HDM-C but not in HDM-P; *TACSTD1* (also known as *EpCAM*), *KRT19* and *CFTR* were modestly suppressed in HDM-H and HDM-P, but especially *CFTR* was actually elevated in HDM-C, and *TACSTD1* and *KRT19* were not affected. Data are expressed as the mean±s.d. (triplicate samples). Statistically significant (**P<0.01, *P<0.05, by Student's *t*-test with comparison to KM as controls). The scale bar, 100 μm.

Expansion of the hFL-HCCs in monolayer (or in spheroids) occurred in serum-free KM. By contrast, serum-free, hormonally defined media (HDM), utilized previously for lineage restriction of hBTSCs to hepatocytes (HDM-H), cholangiocytes (HDM-C) or pancreatic islets (HDM-P)^11^, were used to try to differentiate hFL-HCCs. Cells were monitored for morphological (Fig. 6a) and immunocytochemical changes (Fig. 6b) and were assayed by qRT–PCR for stemness and mature markers (Fig. 6c). Peak levels of stemness genes occurred in KM, whereas those markers were significantly suppressed in all three differentiation HDM. In HDM-C, there was an increase in *CFTR* mRNA and protein. CFTR is found in normal hBTSCs but its levels increase during maturation to cholangiocytes. Higher levels of differentiation were blocked by hFL-HCCs significant production of matrix-degrading factors that resulted in rapid dissolution (within hours) of every matrix substratum tested.

### Cell type of origin for hFL-HCCs revealed by RNA-seq

**Figure 7:**
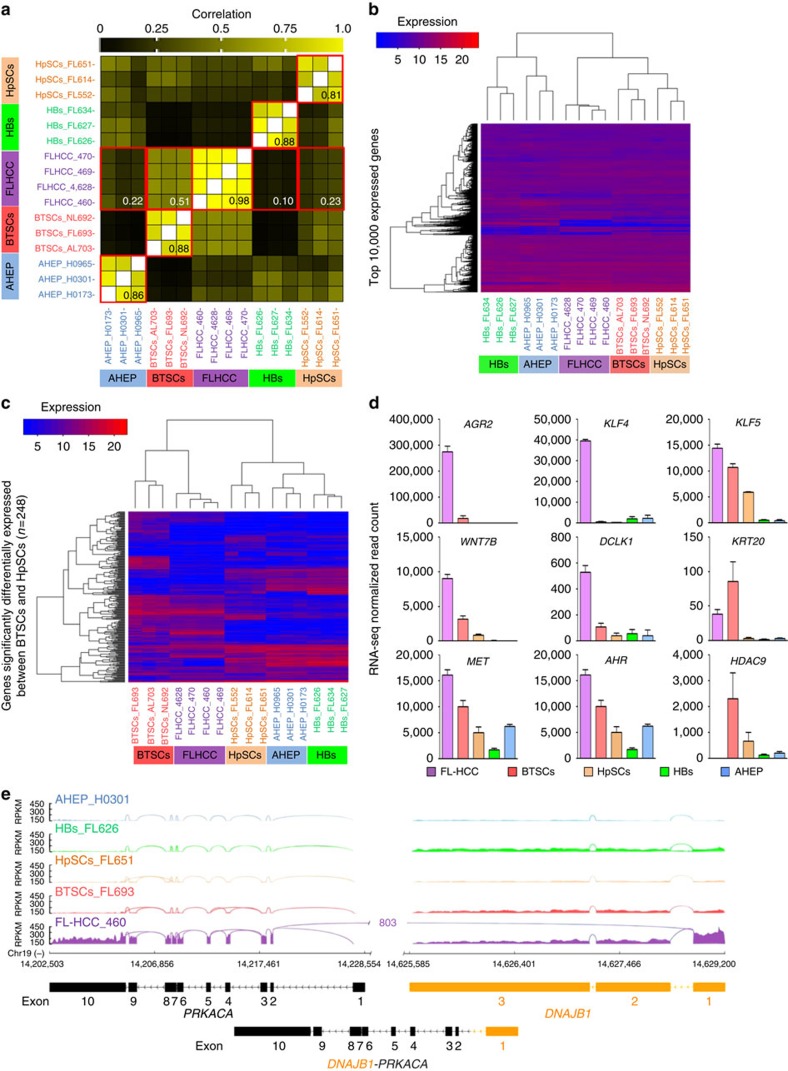
Global gene expression by RNA-seq analyses. (**a**) Correlation heatmap of gene expression profiles from RNA sequencing of hAHEPs, hBTSCs, hHBs and hHpSCs, each from three different donors, as well as hFL-HCC from four tumours in different passaged lines of the transplantable tumour line. Tumour cells were depleted of host-murine cells before being analysed by RNA-seq. Values between 0 and 1 shown in each box correspond to the median pairwise Pearson's correlation coefficient. All genes with an average normalized expected count >50 across all samples were included in the analyses (*n*=14,394). (**b**,**c**) Results of hierarchical clustering analysis based on Euclidian distance of gene expression profiles across the different categories of cells using either the 10,000 most highly expressed genes (**b**) or the 248 genes significantly differentially expressed between hBTSCs and hHpSCs (**c**). For both (**b**) and (**c**), only genes with an average normalized expected count >50 in at least one cell category were considered. (**d**) Histograms of representative genes with distinct expression patterns in hFL-HCCs are shown. (**e**) Sashimi plot of RNA-seq read coverage for fusion gene *DNAJB1-PRKACA*. The *DNAJB1-PRKACA* chimera was detected only in the cells of the hFL-HCC transplantable tumour line and not in any of the normal cell populations. Solid peaks depict reads per kilobase per million reads mapped. Splice/fusion junctions are shown as arcs. The fusion junction joins exon1 of *DNAJB1* with the start of exon2 of *PRKACA*. Four replicate samples of hFL-HCC tumours had 803, 972, 837 and 581 reads, respectively that spanned the fusion junction. We demonstrate that it is not present in normal hBTSCs, hHpSCs, hHBs or hAHEPs.

We performed paired-end high-throughput RNA sequencing in purified populations of sequential maturational lineage stages of human hepatic parenchymal cells: hBTSCs, hepatic stem cells (hHpSCs) and hepatoblasts (hHBs), each from three different donors (Fig. 7). We obtained an average of ∼215 million paired-end reads per sample, of which an average of ∼90% mapped uniquely to the human genome (Supplementary Table 4). We then compared the data with what we had already generated for hAHEPs (*n*=3) and 4 different passages of the hFL-HCC transplantable tumour line. Gene expression profiles were strongly correlated among all samples within each cell type (average pairwise Pearson's *r*^2^=0.98 for the hFL-HCC preparations; 0.88 for hHBs and hBTSCs; 0.86 for hAHEPs; and 0.81 for hHpSCs). The high correlation among the hFL-HCC samples, from early passage (in the first year) to late passage (after 4 years), indicated remarkable stability of gene expression throughout the years of passaging in mice. Cross-category comparisons revealed that gene expression profiles of hFL-HCCs were most strongly correlated with those of hBTSCs (Fig. 7a). This finding was further supported by results of hierarchical clustering analyses, showing that hFL-HCCs are more closely related to hBTSCs than to hHpSCs, hHBs or hAHEPs (Fig. 7b,c and Supplementary Fig. 4).

We also analysed RNA-seq data from all primary hFL-HCC tumours in The Cancer Genome Atlas (*n*=3), as well as from another primary hFL-HCC described by Xu *et al*.^19^ (Supplementary Table 5). We showed that the gene signature of the primary hFL-HCCs more closely resembles that of the hFL-HCC tumour model and of hepatobiliary stem cell populations than that of the more differentiated maturational lineage stages of hepatic parenchymal cells: hHBs and hAHEPs (Supplementary Fig. 5a). Furthermore, the genes and the pathways most significantly altered in the hFL-HCC tumour line relative to hBTSCs are altered in a similar manner in the primary hFL-HCC tumours (Supplementary Fig. 5b–c). These findings further support the stem-cell-rich tumour line as a *bona fide* model of hFL-HCCs.

Unique molecular features of the hFL-HCC tumour line (Fig. 7d) included high expression of *AGR2* (ref. 22) and *PCSK1* (ref. 23), both previously identified as markers of primary hFL-HCCs; *DCLK1* (ref. 24); and *KRT20* (ref. 25), found in endodermal cancers, particularly of intestine; *POU5F1* (also known as *OCT4*) and *KLF4/5*, critical regulators of stemness^26^; and *AHR*, shown to trigger malignant transformation of HpSCs on binding to dioxins and related agonists^27^. *DCLK1* was recently identified as the only marker that accurately distinguishes CSCs from intestinal tumours from normal stem cells^24^. Similarly, we found that *DCLK1* is highly expressed in the CSC-rich hFL-HCC tumour line and primary hFL-HCCs, but lowly expressed in all normal hepatic maturational lineage stages including hBTSCs and hHpSCs. Finally, it is notable that *HDAC9*, which has been linked to tumour suppressive activity through effects on p53 (ref. 28), is most highly expressed in hBTSCs but missing altogether in hFL-HCCs.

Expression data are shown for representative stem/progenitor, hepatocytic, biliary and pancreatic genes, as well as components of the hedgehog signalling pathway, and *HDAC* genes (Supplementary Fig. 6). Results of pathway enrichment analysis for genes differentially expressed in hFL-HCCs compared with hHpSCs or hBTSCs are also shown (Supplementary Fig. 7). Interestingly, the pathways most significantly altered in hFL-HCCs relative to hBTSCs are those that govern lipid and bile acid homoeostasis.

Finally, we detected the *DNAJB1-PRKACA* chimera in the hFL-HCC tumour line and in the primary hFL-HCCs but not in any maturational lineage stage of the normal hepatic parenchymal cells, confirming that it is uniquely expressed in hFL-HCCs. Sashimi plots for the fusion transcript are shown (Fig. 7e).

### The hFL-HCC tumour line most closely resembled hBTSCs

**Figure 8:**
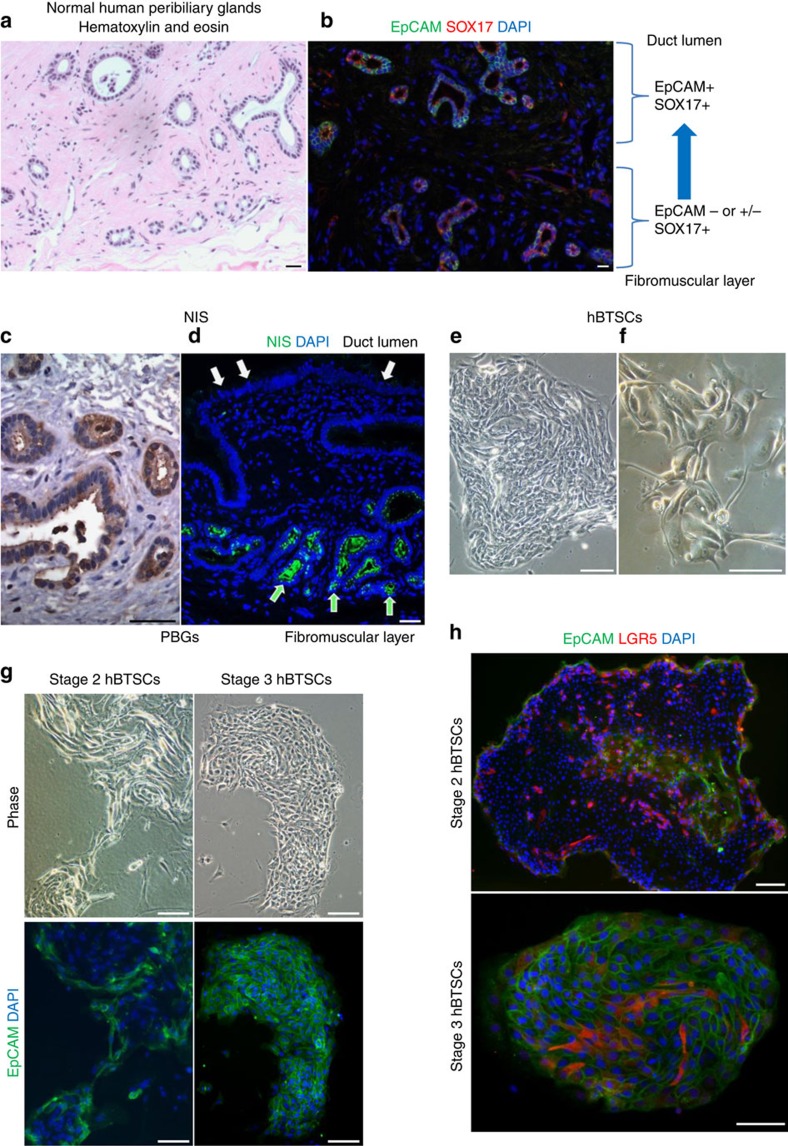
Normal hBTSCs. (**a**) Hematoxylin/eosin stained section of biliary tree demonstrating PBGs found throughout the duct wall (intramural glands). There are others attached by tethers to the bile duct surface (extramural glands). (**b**) Sections stained by IHC for EpCAM and SOX17. Those within the duct wall contain cells of varying phenotypic traits that are found to be in a pattern indicating a radial axis of maturation lineages of the hBTSCs. The most primitive hBTSCs, are located deep within the walls of the bile ducts and near the fibromuscular layers. These cells do not express LGR5 or EpCAM but do express pluripotency genes (for example, OCT4, SOX2, KLF4 and NANOG) and endodermal stem cell markers (for example, SOX9, SOX17 and PDX1). As one moves towards the lumen of the bile duct, the cells gradually lose stem cell traits and acquire mature cell traits. At intermediate stages in this process, the cells acquire LGR5 (stage 2 hBTSCs) and then EpCAM (stage 3 hBTSCS). At the bile duct lumens, no stem cell traits were found but instead only markers of mature cells. (**c**,**d**) NIS, expressed by the primitive hBTSCs (especially those nearest to the fibromuscular layer) but not by mature cells. (**e**,**f**) cultures showing the undulating morphologies of the EpCAM-negative hBTSCs cells. (**g**) Comparison of the two stages of hBTSCs found in culture: those with or without EpCAM expression. (**h**) Stage 2 hBTSCs versus stage 3 hBTSCs with respect to both EpCAM and LGR5. All cultures of hBTSCs were achievable by plating onto culture plastic (or onto hyaluronans) and in serum-free KM. The scale bar, 100 μm.

The finding from the transcriptomic profiling that the hFL-HCCs closely resemble normal hBTSCs caused us to analyse the hBTSCs *in situ* and *in vitro* to learn to what extent there was overlap between hFL-HCCs and particular hBTSCs subpopulations (Fig. 8 and Supplementary Fig. 8). A summary of known phenotypic traits of the hBTSC subpopulations and of the maturational lineages to which they give rise is given in Supplementary Fig. 9 and Supplementary Table 6. Thus far, we have identified three phenotypically distinct hBTSC subpopulations *in situ*, of which two have been observed also in cultures. The distinct hBTSCs are found in peribiliary glands (PBGs) throughout the biliary tree and with gradients in their phenotypic traits depending on their location (Fig. 8a,b)^7^,^8^,^9^,^11^,^13^,^14^. A primary, radial axis of maturation starts with primitive stage 1 hBTSCs, located in PBGs near the fibromuscular layer within the bile duct walls and that express CD44, the hyaluronan receptor, and also the NIS (Fig. 8c,d) but not LGR5 or EpCAM. The PBGs at levels intermediate between the fibromuscular layers and the duct lumen contain cells expressing LGR5 but not EpCAM (stage 2 hBTSCs). Those nearest to the lumen (and also in crypts within gallbladders) express both LGR5 and EpCAM (stage 3 hBTSCs). Finally, at the duct lumens are found only cells that express mature markers and are devoid of stem cell traits.

Two of the three hBTSC subpopulations identified from *in situ* studies correlated with particular types of colonies in culture (Fig. 8e-h). Plating the hBTSCs into serum-free KM and onto culture plastic or onto hyaluronan substrata resulted in cells expressing endodermal transcription factors (for example, SOX9, SOX17 and PDX1), pluripotency genes (for example, OCT4 and KLF4) and LGR5. These were recognized morphologically as colonies of undulating, motile cells with irregular processes. A second category consisted of carpet-like colonies of cuboidal cells with EpCAM on every cell (Fig. 8g,h). Cells devoid of both LGR5 and EpCAM were never observed in culture either because the culture conditions were not adequate or because culturing the cells activated LGR5 expression. The hFL-HCC tumour line had minimal expression of EpCAM and therefore was most similar to the more primitive EpCAM-negative hBTSC subpopulations.

## Discussion

Fibrolamellar hepatocellular cancers are devastating tumours for which the 5-year survival is only 45%; overall mortality is 60%; and half the patients have metastases at the point of diagnosis^1^,^2^,^3^,^4^,^5^. Also concerning is that they are increasing in frequency from unrecognized liver cancers in the 1970s to ∼5% of all liver cancers today^3^,^4^,^5^. As yet, there is no explanation for this increase. Nor is it understood why patients are primarily children to young adults, and more rarely, middle-aged adults, with no prior history of liver disease.

The ability to analyse the variables contributing to the pathogenesis for hFL-HCCs and to search for effective treatments has been severely restricted by the lack of any model system, forcing investigators to deal entirely with primary tumour samples, ones extremely difficult to obtain. Thus, it is of considerable importance that we have succeeded in establishing the first-ever PDX (patient -derived xenograft) model of hFL-HCCs, a transplantable tumour line (not a cell line) that is maintained in immune-compromised hosts such as NSG mice. Prior efforts to produce hFL-HCC tumour lines (or cell lines) have failed. The efforts failed initially with the ascites tumour cells (those that eventually gave rise to the tumour line) when the cells were transplanted immediately into immune-compromised host. Success proved dependent on culture selection in KM, a serum-free medium designed for endodermal stem/progenitors^7^,^11^,^15^,^29^, and on patience to allow the months (more than 6) required for initial tumour formation. Once established, the hFL-HCC cells were able to generate tumours in all (100%) of the animals transplanted. The speed at which the tumours formed was dictated by the number of cells transplanted and was enhanced by use of supplements, particularly hyaluronans, HGF and VEGF.

The hFL-HCC tumour cells were highly invasive and aggressive, albeit slow to form the tumours, since they were able to penetrate through the body wall accessing the peritoneum; when transplanted intraperitoneally, they spread onto every serosal surface. The ability for invasion correlated with the striking ability of the hFL-HCC tumour cells to dissolve every extracellular matrix substratum tested *in vitro*. Other attributes indicating aggressiveness were the tumour's desmoplastic traits^30^,^31^ in which host mesenchymal cells comprised ∼70% of the subcutaneous tumours and >95% of the intraperitoneal tumours, levels higher than those reported for HCCs^32^ or CCAs^33^. Immunoselection to remove host cells resulted in hFL-HCC cells readily cultured as spheroids and with phenotypic traits consistently expressed even after years of passaging in NSG mice. Consistent with reports by others, tumour stroma produced paracrine signals (matrix and soluble signals), important in tumour progression and metastasis^30^,^34^.

IHCs and histology provided evidence for the relationship of hFL-HCC tumour to endodermal stem/progenitors. Histology demonstrated the typical bands of stroma^1^,^2^,^3^,^4^,^5^ surrounding clumps of large tumour cells having prominent nuclei^4^ and aberrations in mitochondria^35^. Co-expression was found for some stem/progenitor markers (for example, OCT4, KLF4 and SHH) and endodermal transcription factors (for example, SOX9 and SOX17), expanding prior findings^1^,^36^. The hFL-HCCs expressed some hepatic traits (for example, HNF4 and HepPar-1), and the remainder expressed endocrine pancreatic traits (for example, PDX1 and PCSK1) or both. Almost all expressed NIS at the protein level, extending prior reports that NIS is expressed in CCAs^37^ and is a target of transcriptional regulation by p53 (ref. 38).

Given the wealth of stem cell traits in the transplantable tumour line, we obtained primary tumour samples of hFL-HCCs from MSKCC to validate the relevance of stemness in this tumour type. Analyses on 27 primary tumours confirmed the expression of some pluripotency genes (for example, OCT4 and KLF4) and endodermal stem cell traits (for example, SOX17 and PDX1) paralleling what was observed with the transplantable tumour line.

A noteworthy feature of the tumour line was its richness in CSCs (>60% CSCs based on flow cytometric analyses of particular markers (for example, CD44+ cells). This is a unique finding given that the average percentage of CSCs in HCCs is ∼0.5–3% (ref. 32) and that in CCAs is ∼10–20% (refs 39, 40). The richness of CSCs in hFL-HCCs was demonstrated functionally by their ability to form tumours in 100% of the mice with as few as 100 cells and by the ease with which they formed spheroids in culture, ones that were maintained for months and were able to be passaged.

Phenotypic properties of hFL-HCCs clinically derive in part from this richness in CSCs and their presumptive origins from hBTSCs, precursors to liver and pancreas. These findings provide clarifications for reports of hFL-HCCs with hepatic, cholangiocytic, and endocrine markers^1^,^2^,^19^,^20^ and, in our studies, intestinal traits. The properties of hFL-HCCs implicate origins from hBTSCs located in PBGs^7^,^8^,^11^ and in crypts at the base of villi within gallbladders^9^. Dramatic evidence of the biliary tree cells as a reservoir of first responders in liver injury and diseases was shown recently using a novel marking method established by Kaneko *et al*.^41^. Lineage tracing studies in mammals^42^,^43^ and zebra fish^44^ indicate that the biliary tree is able to replace the liver following extreme losses of parenchymal cells. In addition, there are growing numbers of reports implicating biliary tree stem/progenitors in liver and pancreatic organogenesis^7^,^8^,^11^. The lineages of stem/progenitors along with adult, diploid parenchymal cells contribute to liver regeneration through formation of liver buds that replace damaged parenchymal cells in diseases such as cirrhosis^45^.

Early stages of malignant transformation of hBTSCs within PBGs have been described by Nakanuma and Sato^46^. More recently, CSC profiles of HCCs and CCAs have been characterized extensively by E.G., D.A. and co-workers and with evidence implicating origins from biliary tree cells for CCAs^40^,^47^. The hFL-HCC tumour line shares properties more with those of CCAs than HCCs.

The TEM studies on the spheroids revealed many noteworthy features, but perhaps the most striking were the electron-dense granules and the extraordinary numbers of mitochondria with abnormal cristae, a condition typical of certain cancers and referred to as oncogenic^48^. This suggests that the mitochondria generated ATP by oxidative phosphorylation and made the cells tolerant of hypoxia. An oncocytic condition with such pleomorphic mitochondria is not known to be associated with HCCs but has been described in pancreatic cancers^49^. The secretory granules are hypothesized to contain factors responsible for the ability of hFL-HCCs to dissolve rapidly (in a few hours) every type of matrix tested as substratum.

The strongest evidence of hBTSCs as the likely origins of hFL-HCCs derives from our RNA-seq studies, which includes analyses of genes across successive maturational lineage stages of normal hepatic cells from hBTSCs to hHpSCs to hHBs to adult hepatocytes (AHEPs). The global transcriptome-wide analyses indicate that hFL-HCCs more closely resemble hBTSCs than the other lineage stages analysed. Also, through RNA-seq analyses we provide independent confirmation that hFL-HCCs uniquely express the *DNAJB1-PRKACA* chimera, a fusion gene coupling the catalytic site of protein kinase A and a heat shock protein^18^,^19^,^20^, resulting in stable activation of protein kinase A. Future investigations are required to explore further this fusion gene and its potential for diagnostic and/or therapeutic utility. To our knowledge, this is the first study to perform global comparative gene expression analysis across four different and successive maturational lineage stages of the normal human biliary tree and hepatic parenchymal cells, including multiple, sequential stages of stem/progenitor cell populations: hBTSCs, hHpSCs and hHBs.

Genetic analyses of hFL-HCCs have been done by Zucman-Rossi and co-workers^50^,^51^, Torbenson *et al*.^2^,^35^,^52^, Honeyman *et al*.^18^, Malouf *et al*.^23^, Xu *et al*.^19^ and, most recently, Lovet and co-workers^20^. They have identified additional biomarkers^53^,^54^, including CD68 (ref. 21), which are distinct from those in other liver cancers^32^,^40^. Earlier studies also indicated that hFL-HCCs have Mosaic G-protein alpha-subunit (*GNAS*)-activating mutations, characterized by STAT3 activation^55^, epidermal growth factor receptor levels higher than in other types of hepatic tumours, and no K-RAS mutations^53^.

The resistance of hFL-HCCs to chemotherapies is probable, given the cells' strong expression of multidrug resistance genes. Their renowned aggressiveness in patients and in immune-compromised hosts correlates with expression of multiple genes, including adhesion molecules (E-cadherin, VCAM-1), matrix receptors (CD44), syndecan-1 (HS-PG) and matrix-degrading factors that include heparanase. Heparanase has been shown to fragment the glycosaminoglycan chains, chemical scaffolds for growth factors and cytokines (for example, FGFs and VEGFs) releasing glycosaminoglycan/growth factor complexes as potent mitogens both at the plasma membrane surface and through processes that transport them to the nucleus^56^,^57^,^58^.

The expression of NIS in most hFL-HCCs suggests that NIS-mediated radio-iodide therapy might be used to treat this malignancy the way it has been—with great success—in treatment of thyroid cancer^59^. However, further studies are ongoing to confirm that NIS expression correlates with functionality and, most importantly, that the anion being transported is, in fact, iodide.

Our findings of remarkably high levels of AHR receptors in hFL-HCCs and in hBTSCs, and a prior report that dioxins preferentially affect hHpSCs^27^, provides clues about possible aetiological factors of hFL-HCCs. AHR agonists have emerged as environmental factors from the plastic industries since World War II, correlating with increased incidence of hFL-HCCs. Further studies are needed to assess this hypothesis.

There is accumulating evidence that there is dysregulation of epigenetic modifications in cancers. This guided us in looking for gene expression of members of the HDAC^60^,^61^ and hedgehog families^62^,^63^. An intriguing finding is the complete loss of *HDAC9* in the hFL-HCC tumour line; the relevance of this to the pathogenic properties of hFL-HCCs is not yet understood. One possibility is that high expression levels of *HDAC9* might be required in normal stem cells, and loss of *HDAC9* might be associated with altered chromatin structure and pathogenesis of hFL-HCCs, possibilities that might suggest novel candidate therapies.

In summary, the hFL-HCCs' richness in CSCs; their probable origins from hBTSCs; the hints that AHR agonists might be aetiological factors; and this first-ever established transplantable tumour line offer novel diagnostic and therapeutic opportunities much-needed for this devastating cancer.

## Methods

### Sourcing of primary tumour samples

Sections (5 μm) prepared from the paraffin blocks of nine primary hFL-HCC tumours and ones from TMAs of 19 normal adult livers and 18 hFL-HCC patients were obtained from MSKCC and used for IHC assays. They were obtained with approval of the Institutional Review Board at MSKCC. Handling of all the samples fully met compliance and privacy requirements as per Health Insurance Portability and Accountability Act (HIPAA).

### Sourcing of normal tissue

Adult, normal, human biliary tissues were dissected from tissue connected to intact livers and pancreases obtained but not used for transplantation into a patient. They were obtained through organ donation programs via United Network for Organ Sharing. Those used for these studies were considered normal with no evidence of disease processes. Informed consent was obtained from next of kin for use of the tissues for research purposes, protocols received Institutional Review Board approval, and processing was compliant with Good Manufacturing Practice. The research protocol was reviewed and approved by the Institutional Review Board for Human Research Studies at the UNC at Chapel Hill, NC, USA.

### Animals

In preliminary studies, several types of immunocompromised mice (for example, athymic nudes, SCID/NODs, NSGs, both male and female and all of them 4–6 weeks of age) were obtained from suppliers or were obtained from breeding colonies on the UNC campus and used as hosts for the hFL-HCC cells. Although tumours developed in all the different immunocompromised mouse strains, the findings were most successful with *NOD.Cg-Prkdc*^*scid*^ *Il2rg*^*tm1Wjl*^*/SzJ*. These are known commonly as NSGs. These mice are devoid of T or B cells, lack functional NK cells and are deficient in cytokine signalling. The strain combines the features of the NOD/ShiLtJ (Stock Number 001976) background, the severe combined immune deficiency mutation (*SCID*, which is caused by a spontaneous mutation in the *Prkdc* gene), and the IL2 receptor gamma chain deficiency. The animals were maintained in the quarters maintained by the Division of Laboratory Animals. Procedures were performed according to protocols approved by the UNC School of Medicine at Chapel Hill Institutional Animal Care and Use Committee. All species were inbred and housed in UNC's Division of Laboratory Animals sterile facility in micro-isolated autoclaved cages with free access to autoclaved water and radiation sterilized food.

### Original handling of the hFL-HCCs used for the tumour line

The tumour used to establish the transplantable tumour line was received as ascites fluid replete with tumour cells. Four litres of ascites fluid were received at UNC within 10 h of removal from the patient. The cells were centrifuged and pooled, yielding ∼2 × 10^7^ cells. They were plated onto plastic or other substrata (laminin, hyaluronans, types I, III or IV collagens) in serum-free KM prepared in either RPMI 1640 or in DMEM-F12 (refs 15, 64) and cultured as two-dimensional monolayers or 3D hydrogels. Serum-free KM has been found to be selective for endodermal stem cells and progenitors^7^,^11^,^29^ and is not permissive for mature cells. Culture selection for tumour cells with stem cell properties in monolayer (two-dimensional) cultures did best on plastic and in KM in DMEM-F12; those in 3D hydrogels behaved similarly in KM prepared in either DMEM-F12 or RPMI 1640, grew more slowly, and, in parallel, caused dissolution of hydrogels by hFL-HCC's enzyme secretions. The culture selection process proved critical for establishment of the transplantable tumour line as clarified in further details below.

### Transplantation of the hFL-HCC cells

Efforts to establish a tumour line by transplanting the original ascites tumour cells immediately after removal from the patient were not successful. Rather, success was achieved only with cells that survived in serum-free KM and on culture plastic or on/in hyaluronans. The original tumour sample consisted of 4 l of ascites tumour cells that were centrifuged, plated onto culture plastic and in serum-free KM for several weeks. Phase images of the original cultures are given in Fig. 5a. The culture-selected tumour cells were transplanted and yielded tumours after an initial passage of more than 6 months in NSG mice. Thereafter, xenografted tumours were passaged by mincing tumour in KM supplemented with 1% hyaluronans (uncross-linked) and further supplemented with 50 ng ml^−1^ each of HGF and VEGF. The tumour mince (∼20 mg) in the KM+1% hyaluronans+growth factors was injected subcutaneously into mice. The tumour mince will form tumours in the absence of hyaluronans and growth factors, but will do so more slowly and will not yield tumours at all in some mice. Consistent, reproducible tumour formation occurred with the use of the supplements. If transplanted intraperitoneally, the tumour spread onto the serosal surfaces throughout the peritoneum and also onto the liver and pancreas.

In passage 8, the tumour cells were dispersed, and the host mesenchymal cells depleted by sorting negatively for cells positive for, H-2 K^d^. The purified hFL-HCC cells were transplanted subcutaneously at cell numbers from 10^2^ to 10^6^. At all concentrations from 10^5^ cells and higher resulted in 100% of the mice forming tumours by 3 months; at 10^3^–10^4^ cells, all formed tumours within 4–5 months. At 100 cells, one tumour formed at 5 months; one at 6 months; and one by 9 months.

Tissue processing of the hFL-HCC tumours to generate cell suspensions for *ex vivo* studies was conducted in RPMI 1640 supplemented with 0.1% bovine serum albumin (BSA), 1 nM selenium and antibiotics. Enzymatic processing buffer contained 600 U ml^−1^ type IV collagenase and 0.3 mg ml^−1^ deoxyribonuclease at 32 °C with frequent agitation for 15–20 min. Enriched suspensions were pressed through a 75 gauge mesh and spun at 1,200 r.p.m. for 5 min before re-suspension. Estimated cell viability by trypan blue exclusion was routinely >95%.

### Culture conditions

All media were sterile-filtered (0.22 μm filter) and kept in the dark at 4 °C before use. Hyaluronans were obtained from Glycosan Biosciences (Salt Lake City, Utah; now part of Biotime, Alameda, CA). Type III and IV collagens and laminin were obtained from Becton Dickenson (RTP, NC).

### Kubota's medium

KM is a serum-free medium designed originally for rodent hepatoblasts^15^ and then found effective also for hHBs and hHpSCs^29^, for hBTSCs^7^, and for pancreatic progenitors^11^. It consists of any basal medium (here being RPMI 1640) with no copper, low calcium (0.3 mM), 10^−9^ M selenium, 0.1% BSA, 4.5 mM nicotinamide, 10^−12^ M zinc sulfate heptahydrate, 10^−8^ M hydrocortisone, 5 μg ml^−1^ transferrin/Fe, 5 μg ml^−1^ insulin, 10 μg ml^−1^ high density lipoprotein and a mixture of purified free fatty acids that are added after binding to purified human serum albumin. The detailed protocol for the preparation of KM has been given in a methods review^64^. KM is now available commercially from PhoenixSongs BIologicals (Branford, CT).

### Hormonally defined media

Supplements can be added to KM to generate a serum-free HDM that will facilitate differentiation of the normal hHpSCs or hBTSCs to specific fates^7^,^11^,^65^. These include supplementation with calcium to achieve at or above 0.6 mM concentration, 1 nM tri-iodothyronine (T3), 0.1 nM of copper, and 20 ng ml^−1^ of basic fibroblast growth factor to generate modified Kubota's Medium (MKM). The medium conditions over and above these and needed to selectively yield hepatocytes (HDM-H) versus cholangiocytes (HDM-C) versus pancreatic islets (HDM-P) are:

1. HDM-H: MKM supplemented further with 7 μg l^−1^ glucagon, 2 g l^−1^ galactose, 10 ng ml^−1^ epidermal growth factor and 20 ng ml^−1^ HGF.
2. HDM-C: MKM supplemented further with 20 ng ml^−1^ VEGF 165 and 10 ng ml^−1^ HGF.
3. HDM-P: The MKM is prepared without glucocorticoids and further supplemented with 1% B27, 0.1 mM ascorbic acid, 0.25 μM cyclopamine, 1 μM retinoic acid, 20 ng ml^−1^ of FGF-7 for 4 days, then changed with one supplemented with 50 ng ml^−1^ exendin-4 and 20 ng ml^−1^ of HGF for 6 more days of induction.

### Magnetic immunoselection of hFL-HCC cells

Human tumour cells were isolated from xenografted tumour as described previously^66^ with some modifications. Negative sorting was done using EasySep magnetic bead immunoselection using the magnetic cups and beads (StemCell Technologies, Vancouver, Canada) and according to the manufacturer instructions. Briefly the dissociated cells were washed in phosphate-buffered saline (PBS) with 3% FBS (staining medium) were treated with FcR blocking antibody and incubated with cocktail of biotin-conjugated anti-mouse antibody against lineage cells (1:10 dilution, Miltenyi Biotech, Auburn, CA) and with biotin-conjugated anti-mouse-MHC class I (H2K^d^ (clone SF1-1.1, 1:100 dilution) and CD31 (clone; MEC13.3, 1:100 dilution) antibodies (BD Biosciences, San Jose, CA) at room temperature for 15 min.

Cells were incubated with biotin selection cocktail for 15 min, and then incubated with magnetic nanoparticles at room temperature for 10 min. The cups were magnetized, and cells or clumps of cells bound to the walls of the cup; those not bound (the human cells) were collected into a separate container. The cells bound to the cups were the mouse cells that were discarded. The human cells were suspended in KM and then plated.

The cells were plated onto culture plastic or on or in hyaluronan hydrogels (some of them supplemented with type III or IV collagen or laminin) and provided with serum-free KM. For the initial plating, the medium was supplemented with 2–5% FBS (HyClone, Waltham, MA). After a few hours, the medium was changed to the serum-free version, and this was used for all subsequent medium changes.

For the cultures of xenografted tumours, the human cells were sorted by immunoselection away from the murine (host) mesenchymal cells and then were plated in serum-free KM from the outset.

### Spheroid formation assays

For spheroid formation assays, 1 × 10^4^ hFL-HCC cells, depleted of host mesenchymal cells by magnetic sorting, were seeded into each well of a six-well plate coated with Ultra-Low Attachment surfaces (Corning, Lowell, MA) and cultured with serum-free KM in the presence or absence of 1 mg ml^−1^ hyaluronans (Sigma, St Louis, MO). For secondary spheroid formation assays, the primary spheroids were collected and then dissociated with NeuroCult Chemical Dissociation Kit (StemCell Technologies, Vancouver, BC, Canada). Cell suspensions were centrifuged at 700 r.p.m. 10 min and resuspended with KM. After 2 weeks, the number of spheroids (>100 μm) were counted.

### Differentiation assays

For these assays, 1 × 10^5^ hFL-HCC cells, depleted of host mesenchymal cells by magnetic sorting, were seeded into each well of a 12-well plate coated with 5 μg cm^−2^ hyaluronan and cultured with KM+2% FBS for overnight. After 16–20 h, the cells were incubated for 7 days with either serum-free KM (as the undifferentiated control) or with serum-free HDM-H, HDM-C or HDM-P. After a total of 7 days culture, cells were harvested for analyses of gene expression.

### Flow cytometric analyses

The dissociated cells were incubated at 4 ^°^C for 30 min with fluorescein isothiocyanate-conjugated or biotin-conjugated anti-mouse-MHC class I (against H2K^d^; clone: 34-1-2S) (eBioscience, San Diego, CA) and anti-human antibodies (see Supplementary Table 7) for cell surface markers. For biotinylated antibody, allophycocyanin-streptavidin (BD Biosciences, San Jose, CA) was used for visualization. The cells were washed with staining medium before analysis. For the intracellular staining of LGR5, the cells were incubated with antibodies against the cell surface antigens as usual, and then, were fixed with 4% paraformaldehyde (PFA)/PBS at 4 ^°^C for 20 min. After washing with staining medium, the cells were resuspended in permeabilization buffer (PBS with 1% foetal calf serum, 0.1% sodium azide, and 0.1% saponin) with PE-conjugated anti-LGR5 antibody at 4 ^°^C for 30 min. Antibodies used are listed in Supplementary Table 7. The labelled cells were washed with permeabilization buffer, and then analysed by FACSCalibur (BD Biosciences).

### Immunohistochemistry and immunocytochemistry

For immunofluorescent staining, 5 μm frozen sections or cultured cells were fixed with 4% PFA for 20 min at room temperature, rinsed with PBS, blocking with 10% goat serum in PBS for 2 h, and rinsed. Fixed cells were incubated with primary antibodies at 4 °C for 14 h, washed, incubated for 1 h with labelled isotype-specific secondary antibodies, washed, counterstained with 4′,6-diamidino-2-phenylindole for visualization of cell nuclei and viewed using Leica DMIRB inverted microscope (Leica, Houston, TX) or a Zeiss ApoTome Axiovert 200M (Carl Zeiss, Thornwood, NY).

For immunohistochemistry, the tissues were fixed in 4% PFA overnight and stored in 70% ethanol. They were embedded in paraffin and cut into 5 μm sections. After deparaffinization, antigen retrieval was performed with sodium citrate buffer (pH 6.0) or EDTA buffer (pH 8.0) in a steamer for 20 min^67^. Endogenous peroxidases were blocked by incubation for 15 min in 3% H_2_O_2_. After blocking, primary antibodies reacting against human but not mouse cells and were applied at 4 °C overnight. M.O.M immunodetection kit (Vector Laboratories, Burlingame, CA) was used for detecting primary mouse anti-human antibodies on mouse xeno-transplanted hFL-HCC tumour to avoid the inability of the anti-mouse secondary antibody to endogenous mouse immunoglobulins in the tissue. Sections were incubated for 30 min at room temperature with ImmPRESS peroxidase-micropolymer staining kits and 3,3'-diaminobenzidine substrate (Vector Laboratories, Burlingame, CA). Sections were lightly counterstained with hematoxylin. Antibodies used are listed in Supplementary Table 8. Control images are given in Supplementary Fig. 2a.

### IHC analyses for NIS

Preparation of sections was as given above. Antigen retrieval was performed by incubating with boiling 10 mM citrate buffer (pH 6.0) for 15 min. Endogenous peroxidase activity was quenched by incubating with 3% H_2_O_2_ in methanol for 10 min. Tissue sections were blocked for 1 h with 5% goat serum in PBS, (137 mM sodium chloride, 2.7 mM potassium chloride, 4.3 mM sodium phosphate (dibasic, anhydrous), 1.4 mM potassium phosphate (monobasic, anhydrous), pH 7.4). Slides were incubated for 2 h with (or without, for negative controls) a, site-directed polyclonal antibody against the carboxy-terminus of human NIS^68^. The initial concentration of the antibody was 0.5 μg μl^−1^ and was diluted in 0.5% BSA in PBS 1:5,000. All slides were incubated for 10 min with anti-rabbit poly-HRP conjugated antibody (SuperPicture, Zymed Laboratories). Slides were monitored after addition of the chromogen solution until the peroxidase reaction took place. Tissue sections were counterstained with hematoxylin, dehydrated, mounted and dried overnight. Immunoreactivity was analysed by light microscopy.

### Transmission electron microscopy

The hFL-HCC spheroids were fixed with 3% glutaraldehyde in 0.15 M sodium phosphate buffer, pH 7.4, for 1 hour at room temperature and stored at 4 °C until processed. Following three rinses with 0.15 M sodium phosphate buffer, pH 7.4, the samples were post-fixed for 1 h with 1% osmium tetroxide/1.25% potassium ferrocyanide/0.15 M sodium phosphate buffer, pH 7.4, followed by rinses in deionized water. The spheroids were dehydrated using increasing concentrations of ethanol (30, 50, 75 and 100% for 10 min each) and two changes of propylene oxide (15 min each). Following infiltration overnight in a 1:1 mixture of propylene oxide/Polybed 812 epoxy resin (Polysciences, Warrington, PA) and 24 h in 100% resin for 24 h, the spheroids were embedded in fresh Polybed 812 epoxy resin. The spheroids were sectioned transversely at 70 nm using a diamond knife and a Leica Ultracut UCT microtome (Leica Microsystems, Wetzlar, Germany). Ultrathin sections were mounted on 200 mesh copper grids and stained with 4% aqueous uranyl acetate and Reynolds' lead citrate. The grids were observed at 80 kV using a LEO EM910 TEM (Carl Zeiss SMT, LLC). Digital images were taken using a Gatan Orius SC 1000 CCD Camera with DigitalMicrograph 3.11.0 software (Gatan, Pleasantan, CA).

### The qRT–PCR assays

For the quantitative reverse transcription polymerase chain reaction (qRT–PCR) assays, total RNA was extracted from the cells using RNeasy Micro Kit or RNeasy Mini Kit (Qiagen GmbH, Valencia, CA). First-strand cDNA synthesized using the Primescript first-strand cDNA synthesis kit (Takara, Otsu, Japan) was used as a template for PCR amplification. Quantitative analyses of mRNA levels were performed using Power SYBR Green PCR Master Mix with Applied Biosystems 7500 Real-time PCR System (Applied Biosystems, Foster City, CA). The primers were annealed at 50 °C for 2 min and 95 °C for 10 min, followed by 40 cycles of 95 °C (15 s) and 60 °C (1 min). Expression of glyceraldehyde-3-phosphate dehydrogenase was used as a control standard. Primer sequences are listed in Supplementary Table 9.

### RNA sequencing and gene expression analysis

RNA was purified using Qiagen RNeasy Kit from hAHEP, hHBs, hHpSCs, and hBTSCs, each from three different donors, as well as four passages of the hFL-HCC tumour line from the same donor. RNA integrity analysis was performed using an Agilent 2000 Bioanalyzer. The cDNA libraries were generated using the Illumina TruSeq Stranded mRNA preparation kit and sequenced on the Illumina HiSeq 2500 platform. Two samples were sequenced per lane, occupying a total of 8 lanes for all of the samples (one flow cell). Quality control analysis was completed using FastQ. Mapping of sequence reads to the human genome (hg19) was performed with MapSplice2 using default parameters. Transcript quantification was carried out by RSEM analysis, and DESeq was used to normalize gene expression and identify differentially expressed genes. MapSplice2 was also used to detect candidate fusion transcripts. Fusion calls were based on the depth and complexity of reads spanning candidate fusion junctions. Gene expression profiles were compared using Pearson's correlation analysis and hierarchical clustering was performed in R. Hierarchical clustering was performed following Variance Stabilizing Transformation provided in the DESeq package. Pathway enrichment analysis was performed with the Ingenuity Pathway Analysis software. Differential gene expression analysis was conducted only on genes with a minimum average normalized count >50 in at least one category.

### Statistical analysis

Statistically significant differences between samples are calculated by using Student's two-tailed *t*-test and results are presented as the mean±s.d. *P* values of <0.05 were considered statistically significant.

## Additional information

**Accession codes:** The hFL-HCC RNA-Seq data set has been deposited in the Gene Expression Omnibus (GEO) database under accession code GSE73114

**How to cite this article:** Oikawa, T. *et al*. Model of fibrolamellar hepatocellular carcinomas reveals striking enrichment in cancer stem cells. *Nat. Commun.* 6:8070 doi: 10.1038/ncomms9070 (2015).

## Supplementary Material

Supplementary Information Supplementary Figures 1-9, Supplementary Tables 1-9, Supplementary Notes 1-3 and Supplementary References

## Figures and Tables

**Table 1:** Limiting dilution tumourigenicity assays of hFL-HCC cells in NSG mice.

**Tumour cells injected**	**Passage #**	**# of cells injected**	**# mice with tumours/# mice injected**	
			**3 months**	**⩾4–5 months**
Cell suspension comprised of a mixture of host (murine) and human FL-HCC cells	P1*	2 × 10^7^	0/1	1/1 (>6 months)
	P2	∼3 × 10^7^	2/2	2/2
	P3	∼3 × 10^7^	7/9	9/9
	P4	∼3 × 10^7^	6/12	12/12
	P5	∼3 × 10^7^	4/9	9/9
	P6	∼3 × 10^7^	3/20	19/20
	P7	∼3 × 10^7^	12/41	41/41
Human FL-HCC cells depleted of host (murine) cells	P8	1 × 10^5^	5/5	5/5
	P8	1 × 10^4^	4/5	5/5
	P8	1 × 10^3^	2/5	5/5
	P8	1 × 10^2^	0/3	3/3

FL-HCC, fibrolamellar hepatocellular carcinomas; HGF, hepatocyte growth factor; KM, Kubota's medium; VEGF, vascular endothelial cell growth factor.

^*^P1 was comprised of donor ascites cells, cultured selected in serum-free KM and then injected. P2–P7 were minced tumours comprised of ∼65% host mesenchymal cells and ∼35% human tumour cells (the # of cells injected is estimated to be the number of human tumour cells); P8 was done with immunoselected (sorted) human cells depleted of murine mesenchymal cells. In the early passages, it was found that the length of time needed for tumour formation was reduced significantly, from 4–5 months to 2–3 months, by transplanting the tumour cells with supplements comprised of hyaluronans, HGF and VEGF; the supplements became part of the standard protocol for the transplantation.

**Table 2:** Summary of IHC assays on primary tumour samples.

**Original block samples**		**TMA samples**		
**Antigen**	**hFL-HCC**	**Antigen**	**Adult liver**	**hFL-HCC**
BMI1	4/9	SOX9	0/19	12/18
SOX9	7/9	SOX17	0/19	8/18
PDX1	7/9	PDX1	0/19	13/18
NIS	7/9	OCT4	0/19	7/18
SHH	9/9	SALL4*	0/13	3/6
HepPar-1	5/6	SHH	0/19	18/18

hFL-HCC, human fibrolamellar hepatocellular carcinomas; SHH, sonic hedgehog; NIS, sodium iodide symporter; TMA, tissue microarray.

^*^With SALL4 staining, some paraffin sections were lost owing to the buffer conditions used for antigen retrieval.
