# Supplementary material for: Model of fibrolamellar hepatocellular carcinomas reveals striking enrichment in cancer stem cells
Source: Nat Commun. 2015 Oct 6;6:8070. doi: 10.1038/ncomms9070 (PMC4600730; doi:10.1038/ncomms9070)
Supplement: Supplementary Information — Supplementary Figures 1-9, Supplementary Tables 1-9, Supplementary Notes 1-3 and Supplementary References [file ncomms9070-s1.pdf]

IHC assays on the original hFL-HCC patient's ascites fluid

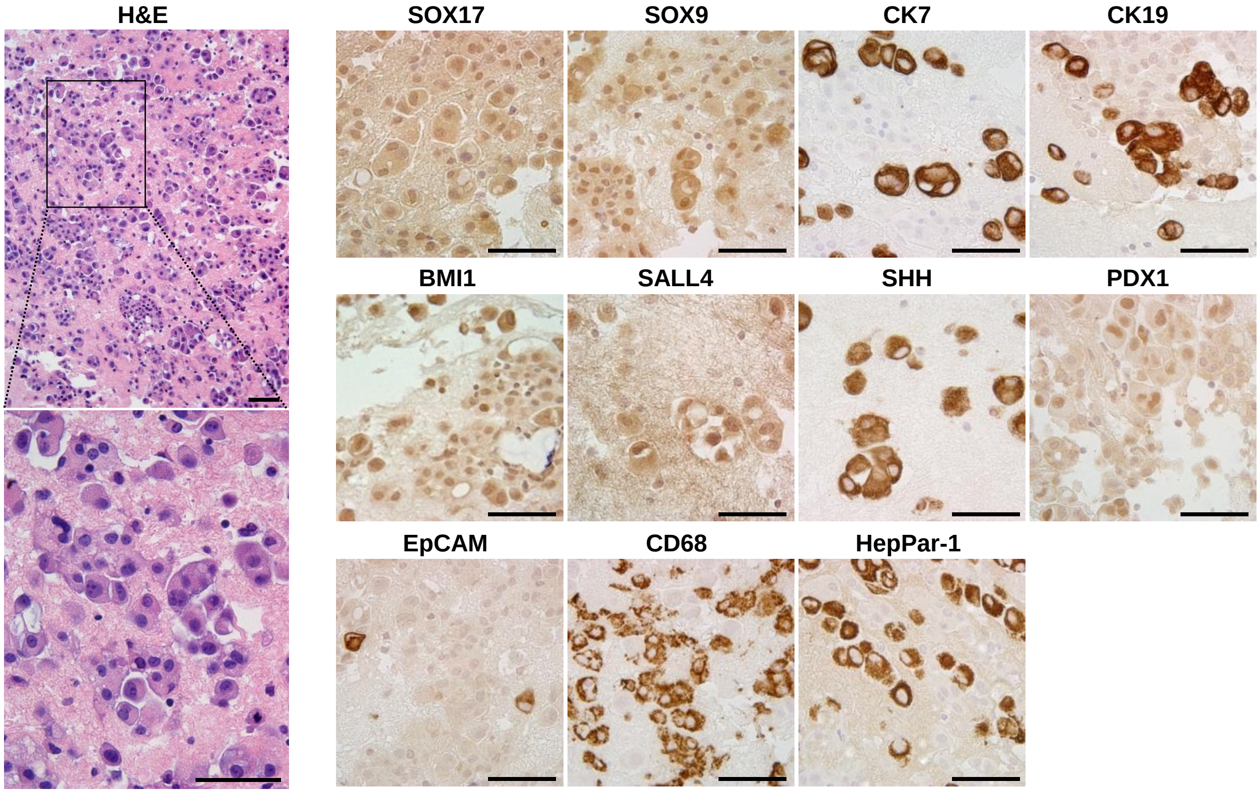

Supplementary Figure. 1. Cytology and IHC assays on cytospun ascites tumor cells. Cytology revealed small aggregates of tumor cells with large pleomormphic nuclei and some forming partial ductular structures. The IHC assays indicated strong positivity for endodermal stemness markers (SOX17, SOX9, PDX1, SALL4, and BMI1), hepatic markers (HepPar-1, CK7 and CK19), and other markers (SHH and CD68). The scale bar = 50 μm.

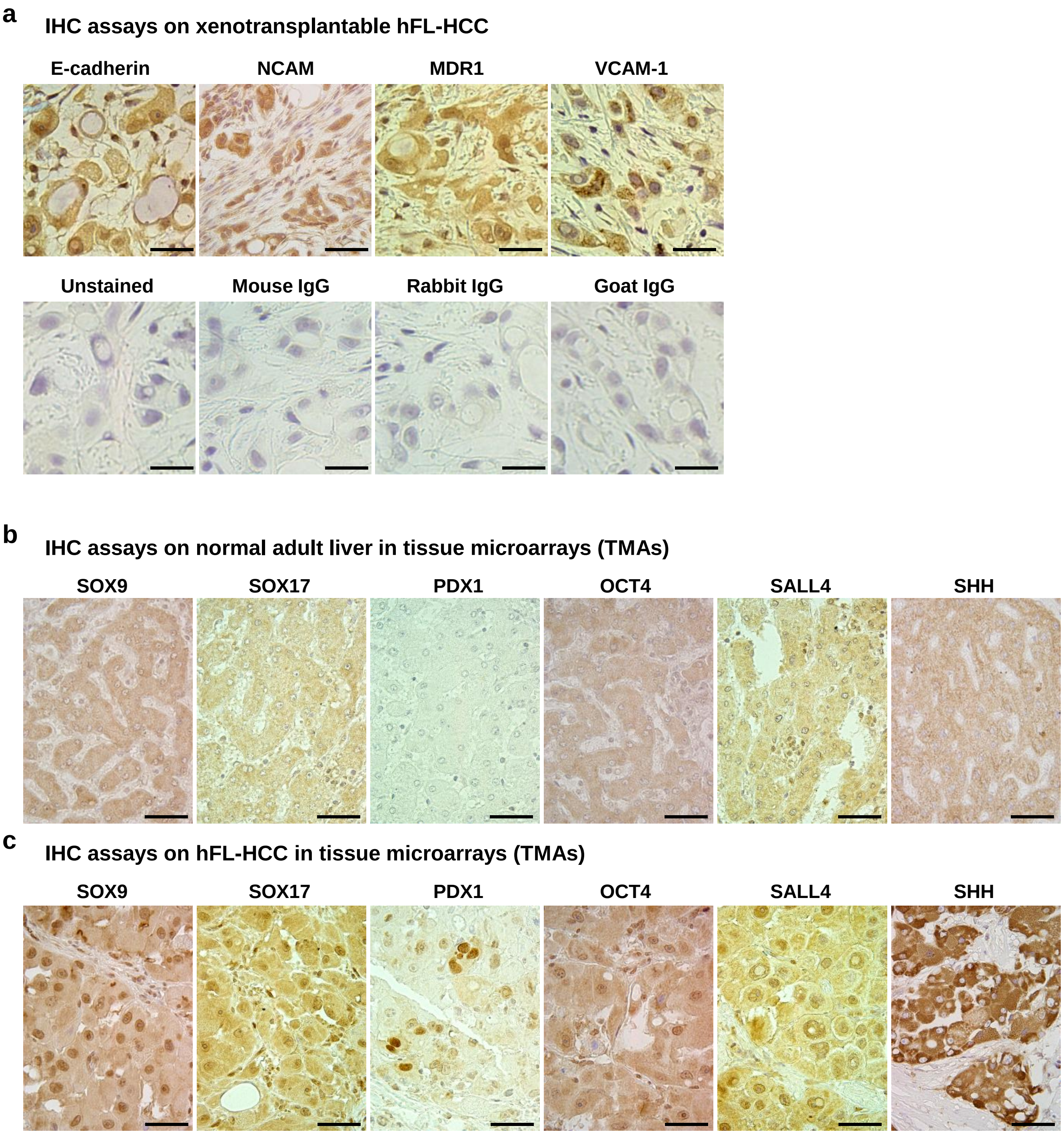

Supplementary Figure. 2. IHC assays on the xenotransplantable tumor line (Tu-2010), and clinical tissue microarray samples of 18 human FL-HCCs versus 19 normal livers. (**a**) Additional IHC assays of the xenotransplantable tumor. Other markers found to be positive included E-cadherin, NCAM, two forms of multidrug resistance gene (MDR1), and VCAM-1. Controls for the IHC assays are also provided. (**b**) Tissue microarrays (TMAs) of human normal adult liver versus (**c**) human fibrolamellar hepatocellular carcinoma (hFL-HCC). The scale bar = 25 μm.

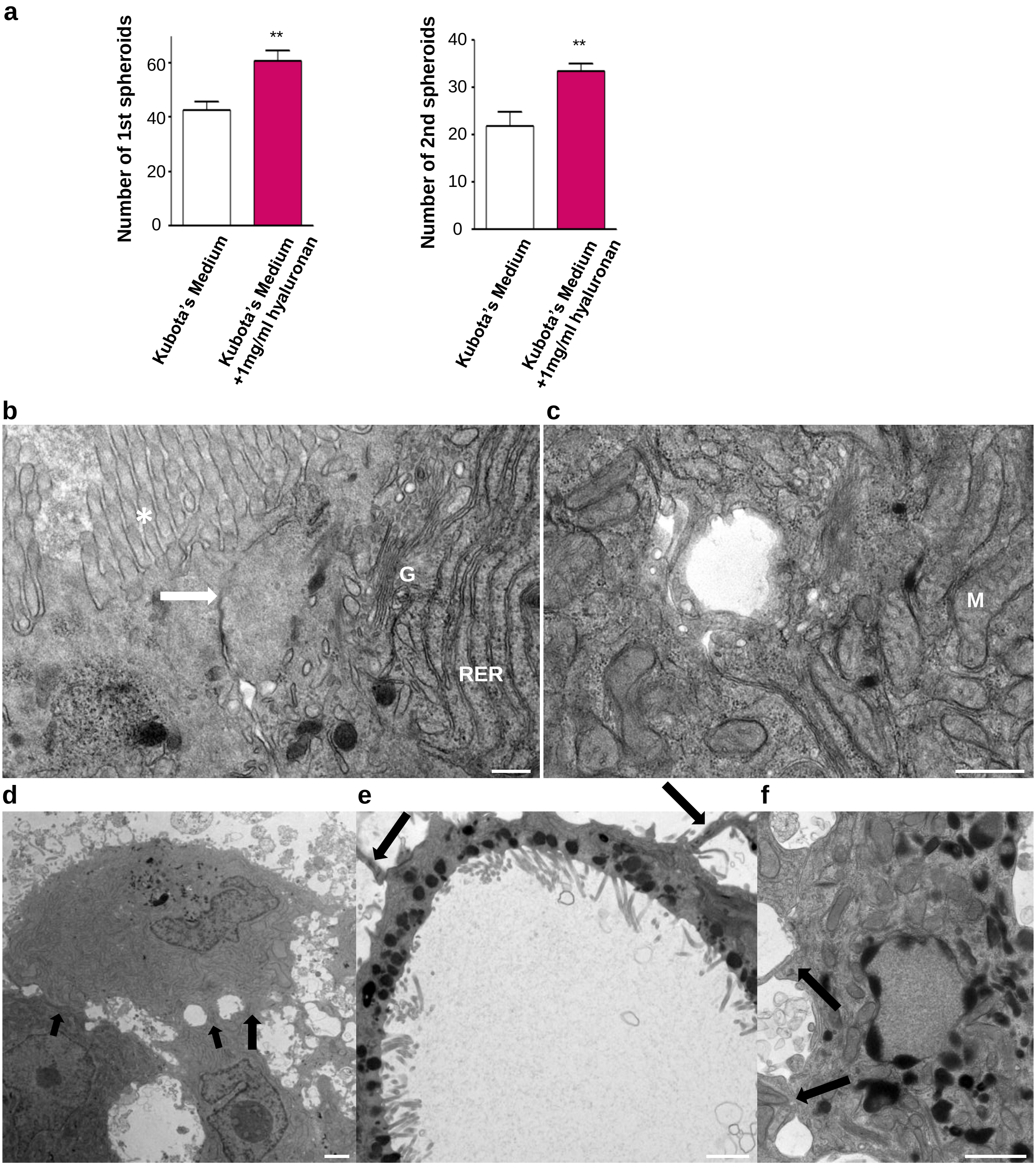

Supplementary Figure. 3. Spheroid cultures. (**a**) The addition of 1mg/ml hyaluronan significantly enhanced the formation of hFL-HCC spheroids. Data are represented as the mean spheroids (>100 μm) count ± SD (triplicate samples). Statistically significant (**p<0.01, by Student's t test with comparison to Kubota's Medium as control). (**b, c**) Tumor cells displayed numerous microvilli at their apical pole (asterisk) and tight junctions (arrow) at cell to cell contact, meaning the tumor cells could still polarize. Cells were rich in rough endoplasmic reticulum (RER) and Golgi apparatus (G) and a wealth of mitochondria (M) with aberrant cristae. (**d**, **e**, **f**) Tumor cells were connected with tunneling nanotubes (TNT: arrows in e); Filopodia-like protrusions, which proceed TNT formation, were present and established physical contact with neighboring cells (arrows in d and e). The scale bar correlates with different lengths in the different images: The scale bar = 200 nm (**b**-**c**), 1 μm (**d**-**f**)

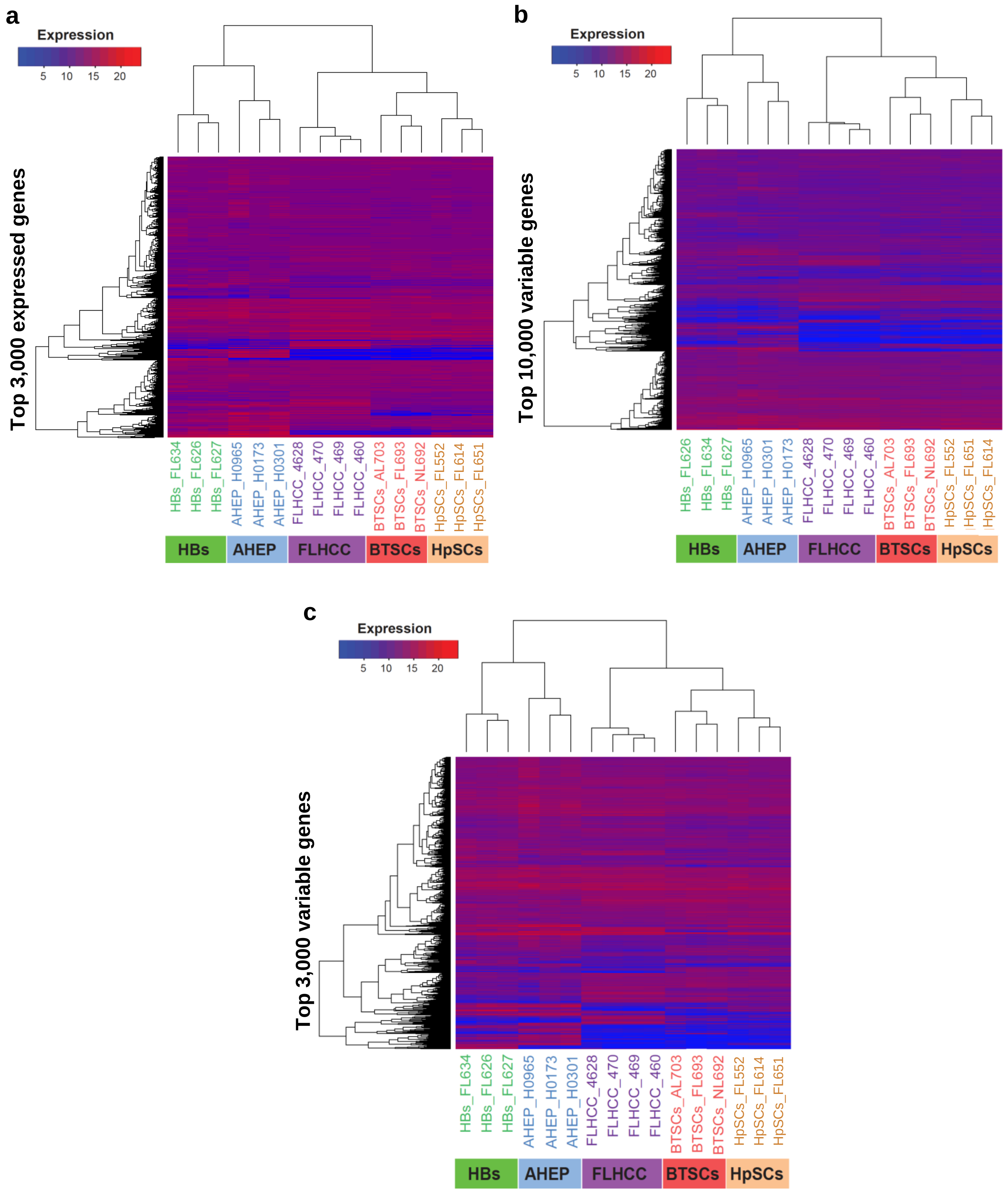

Supplementary Figure. 4. Hierarchical clustering analysis of RNA-seq data from lineage stages of the liver and the hFL-HCC tumor line. Clustering analysis was based on Euclidean distance and complete linkage and performed using the 3,000 most highly expressed genes (**a**), the 10,000 most variable genes (**b**), or the 3,000 most variable genes (**c**). All genes used had an average expected normalized count > 100 across all samples.

a

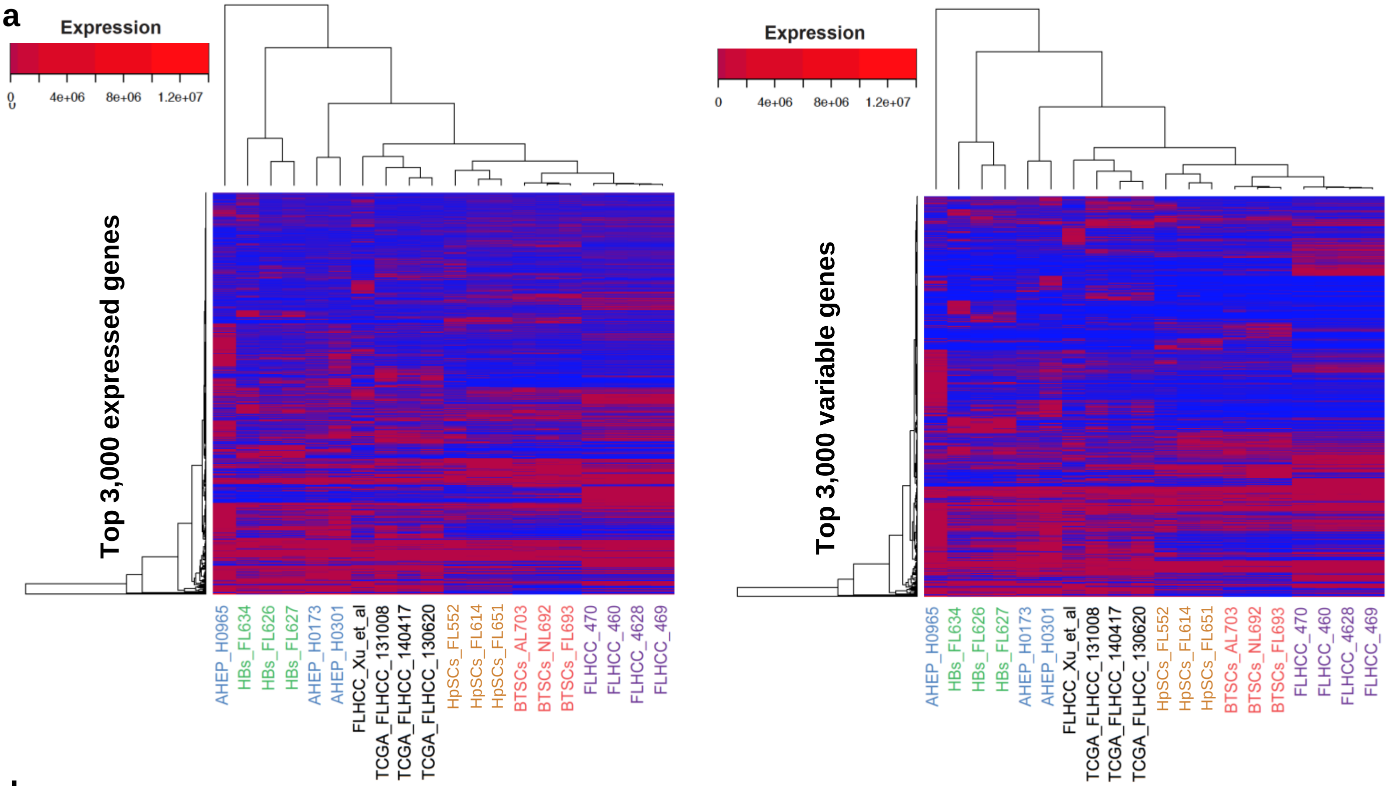

b

IPA Analysis

**Primary FL-HCC vs. BTSCs (n = 1,308)**	**FL-HCC tumor line vs. BTSCs (n = 1,692)**
FXR/RXR Activation (4.80E-37)	FXR/RXR Activation (2.15E-11)
LXR/RXR Activation (1.68E-28)	LXR/RXR Activation (4.17E-08)
Complement System (2.02E-22)	Acute Phase Response Signaling (9.12E-08)
Coagulation (7.48E-19)	LPS/IL-1 Mediated Inhibition of RXR Function (1.45E-07)
Acute Phase Response Signaling (7.86E-18)	Axonal Guidance Signaling (2.69E-07)

c

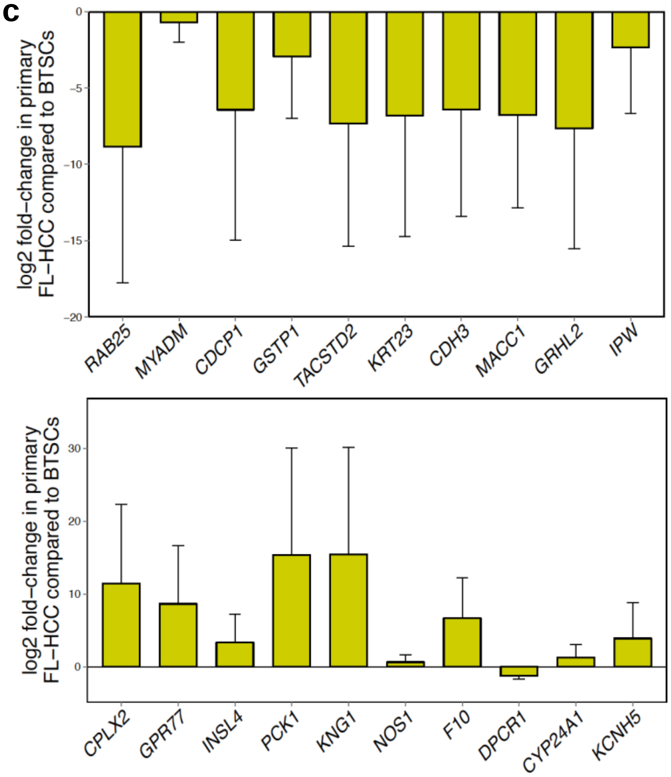

Supplementary Figure. 5. The hFL-HCC tumor line is representative of primary hFL-HCCs. (**a**) Hierarchical clustering analysis of maturational lineage stages of the liver (hBTSCs, hHpSCs, hAHEP), the hFL-HCC tumor line (TU-2010), and primary hFL-HCCs (3 from TCGA, 1 from Xu et. al.[19]) based on Euclidean distance and Ward's method without variance stabilizing transformation and performed using the 3,000 most highly expressed (left) or the 3,000 most variable genes (right). (**b**) Results of Ingenuity Pathway Analysis (IPA) for genes significantly differentially expressed between primary hFL-HCC and hBTSC (left) as well as the hFL-HCC tumor line and hBTSC (right). P-values are shown in parentheses. (**c**) The top 10 most significantly down-regulated (top) and up-regulated genes (bottom) in the hFL-HCC tumor line compared to hBTSCs were determined and their expression in primary hFL-HCCs relative to hBTSCs (log2 fold change) are shown. Error bars show standard deviation.

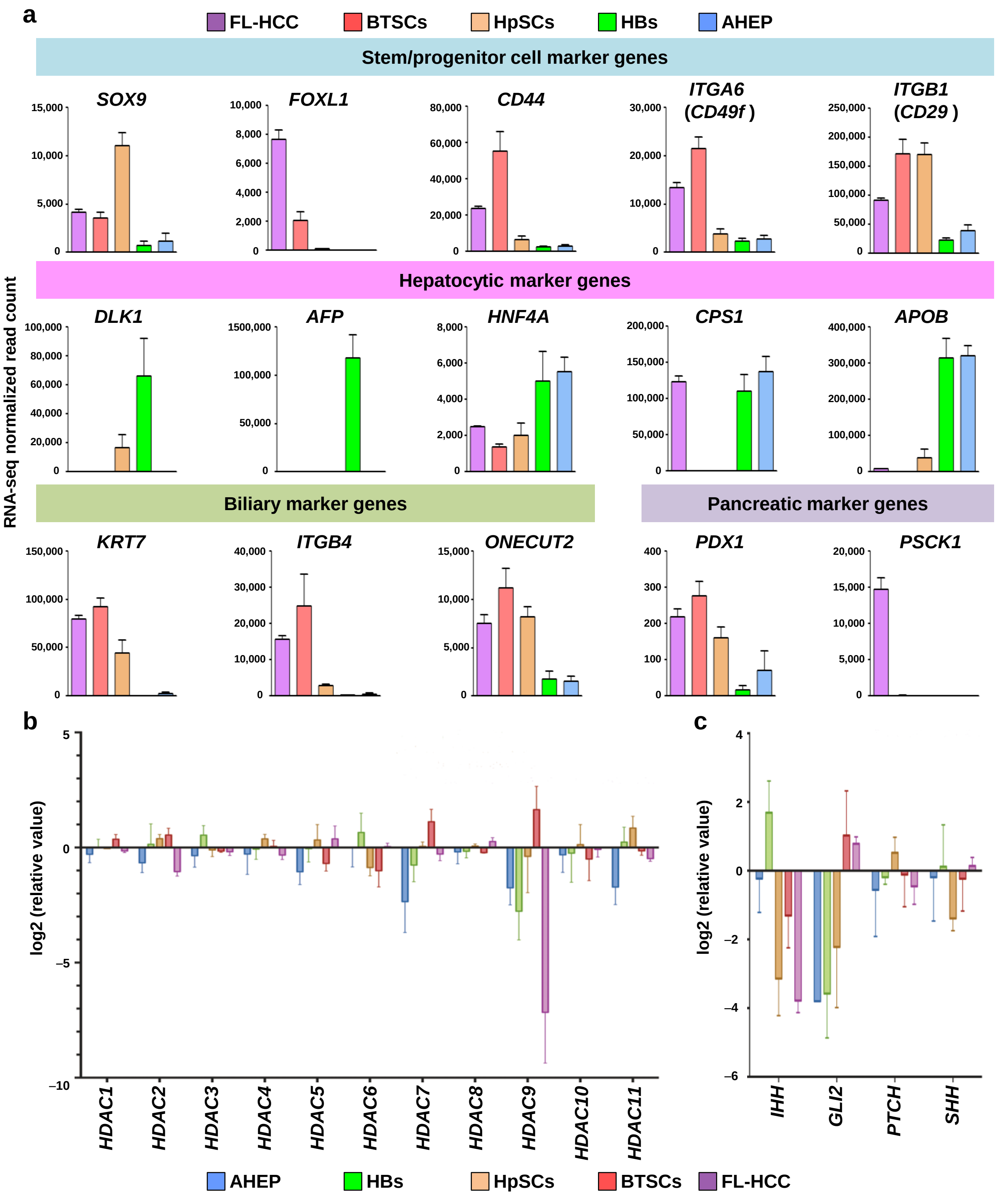

Supplementary Figure. 6. Expression data for representative cell type marker genes. (**a**) RNA-seq normalized expected count data shown across all cell types for genes that have previously been reported as markers of stem cells/progenitors (*SOX9, FOXL1, CD44, ITGA6* [*CD49f*] and *ITGB1* [*CD29*]), hepatocytes (*DLK1*, *AFP*, *HNF4A*, *CPS1*, and *APOB*), biliary tree (*KRT7*, *ITGB4* and *ONECUT2*), and pancreas (*PDX1* and *PCSK1*). (**b**) Expression data for genes encoding histone deacetylases. Log_2 relative expression value shown across all cell types for genes that code for histone deacetylases. Histone deacetylase 9 (*HDAC9*) is lost entirely in hFL-HCCs. (**c**) Expression data for genes in the hedgehog signaling pathway. Log_2 relative expression value shown across all cell types for genes in the hedgehog signaling pathway. Error bars represent standard error of the mean.

IPA analysis

FL-HCC vs. BTSC (n=1692)	FL-HCC vs. HPSC (n=1783)	BTSC vs. HPSC (n=248)
FXR/RXR Activation (2.15E-11)	Coagulation System (3.98E-10)	FXR/RXR Activation (7.94E-30)
LXR/RXR Activation (4.17E-08)	Acute Phase Response Signaling (8.32E-10)	LXR/RXR Activation (2.00E-22)
Acute Phase Response Signaling (9.12E-08)	LXR/RXR Activation (8.71E-09)	Coagulation System (1.00E-18)
LPS/IL-1 Mediated Inhibition of RXR Function (1.45E-07)	Hepatic Fibrosis / Hepatic Stellate Cell Activation (8.32E-08)	Acute Phase Response Signaling (3.98E-18)
Axonal Guidance Signaling (2.69E-07)	FXR/RXR Activation (1.10E-07)	Atherosclerosis Signaling (7.94E-14)
Granulocyte Adhesion and Diapedesis (3.02E-07)	LPS/IL-1 Mediated Inhibition of RXR Function (2.14E-07)	Extrinsic Prothrombin Activation Pathway (7.24E-10)
Role of Tissue Factor in Cancer (1.02E-06)	Nicotine Degradation II (8.13E-05)	Clathrin-mediated Endocytosis Signaling (4.27E-08)
Hepatic Cholestasis (2.88E-06)	Axonal Guidance Signaling (8.91E-05)	IL-12 Signaling and Production in Macrophages (7.94E-08)
Leukocyte Extravasation Signaling (4.57E-06)	Serotonin Degradation (1.00E-04)	Intrinsic Prothrombin Activation Pathway (1.07E-06)
Xenobiotic Metabolism Signaling (7.42E-06)	Atherosclerosis Signaling (1.66E-04)	Complement System (2.43E-06)
Atherosclerosis Signaling (8.71E-06)	Thyroid Hormone Metabolism II (via Conjugation and/or Degradation) (1.70E-04)	Production of Nitric Oxide and Reactive Oxygen Species in Macrophages (6.46E-05)

Top 10 results for IPA analysis. Gene lists for IPAs are DE expressed from DESeq (FDR P<0.05) and average expression in one of the disease categories > 50. BTSC vs. HPSC only lists 9 because the rest of the results are just from the same two genes (see IPA output spreadsheet).

Supplementary Figure. 7. Pathway enrichment analysis. Results of ingenuity pathway analysis (IPA) are shown for genes significantly differentially expressed between human FL-HCC (hFL-HCC) and human biliary tree stem cells (hBTSCs), hFL-HCCs and human hepatic stem cells (hHpSCs), and hBTSCs and hHpSCs.

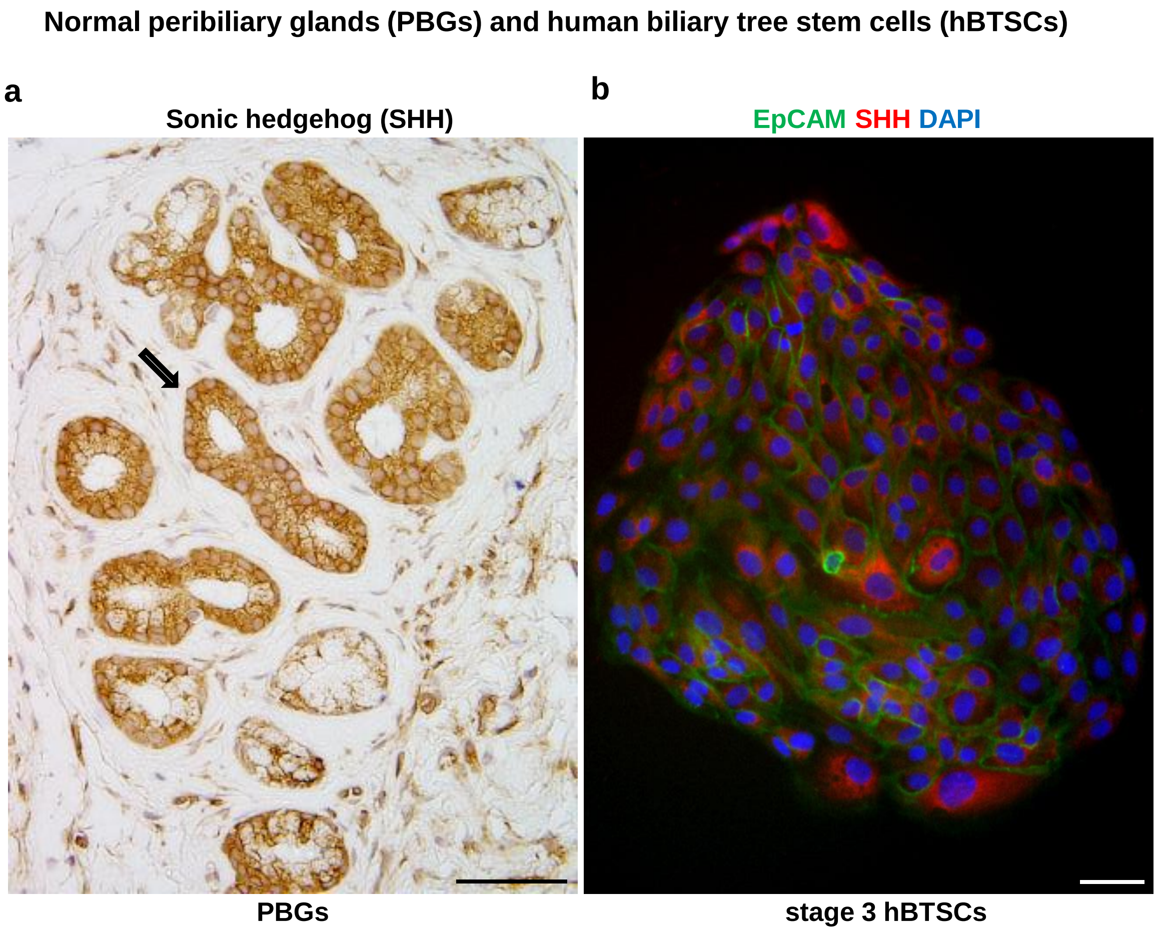

Supplementary Figure. 8. Sonic hedgehog expression in normal peribiliary glands and hBTSCs. (**a**) Expression of sonic hedgehog (SHH) in normal peribiliary glands (PBGs: arrow). (**b**) Sonic hedgehog (red) and EpCAM (green) expression in stage 3 biliary tree stem cells (hBTSCs). The scale bar = 100 μm.

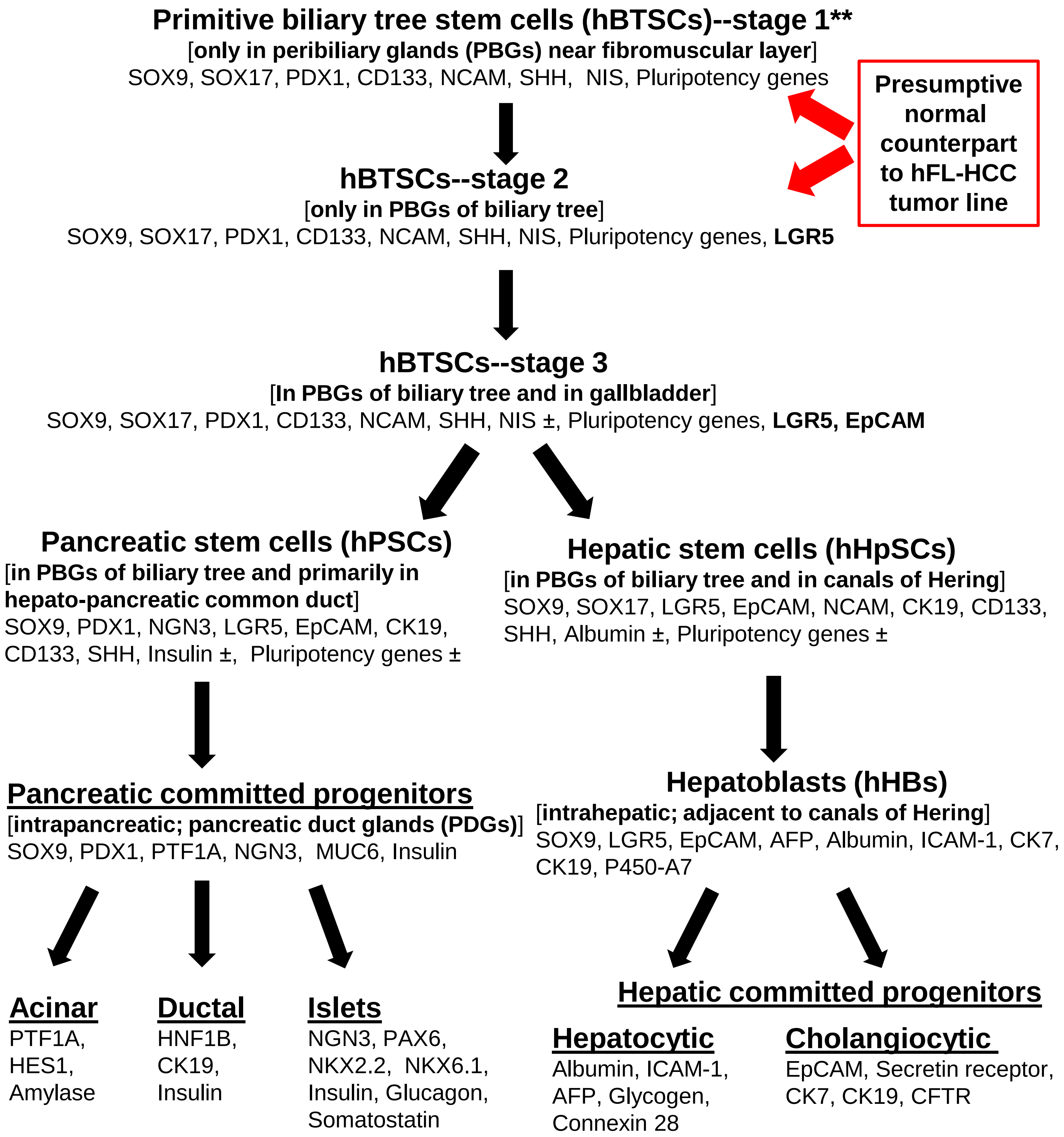

Supplementary Figure. 9. Chart of known biliary tree stem cell populations and their lineage connections and proposal as to for the normal counterpart to the hFL-HCC tumor cells from the transplantable tumor line. The hFL-HCC tumor cells are essentially negative for EpCAM expression. The normal counterparts are likely to be stage 2 BTSCs. However, since all culturing of the hBTSCs is correlated with LGR5 expression, and since we do not know if culturing of the cells activates LGR5, there is the possibility that the tumor line is actually derived from stage 1 BTSCs.
Pluripotency genes: SOX2, OCT4, KLF4, NANOG, TROP-2, BMI1 and SALL4. Cells at all stages express CK8 and CK18. **NIS**= sodium iodide symporter.
Stage 1 hBTSCs have been observed *in situ* in the biliary tree but have **not been successfully cultured under the conditions tested. hBTSCs surviving in culture in serum-free, Kubota's Medium and on plastic or on hyaluronans are either stage 2 or stage 3 hBTSCs.

Biomarkers	Assays	Staining Intensity (% Cells Staining) or Gene Expression Change	Comments
Ribonucleotide reductase subunit M1 (RRM1)	IHC	2+ (80%)	Certain drugs, such as gemcitabine, are of little benefit due to high expression of RRM1
Breast cancer resistant protein (BCRP)	IHC	2+ (75%)	Obviates usefulness of cisplatin and carboplatin
Secreted protein acidic and rich in cysteine (SPARC)	IHC	2+ (35%)	Nab-paclitaxel
SPARC	Microarray	Increased (3.51)	Nab-paclitaxel
Multidrug resistance associated protein 1 (MRP1)	IHC	2+ (30%)	Minimal effects expected with etoposide, vincristine
ABCC1	Microarray	Increased (3.03)	Minimal benefit with paclitaxel, topotecan
HER2/Neu	IHC	2+ (10%) and 1+ (30%)	FISH analyses were done, and 60 interphase nuclei were examined; the ratio of HER2/Neu signals to chromosome 17 signals was 1.63 to 1 indicating no amplification of this gene
HIF1A	Microarray	Increased (10.66)	Agents associated with clinical benefits include sorafenib, sunitinib, bevacizumab
PDGFRB	Microarray	Increased (2.3)	Agents associated with clinical benefits include sorafenib, sunitinib,imatinib
TOP2B	Microarray	Increased (4.55)	Beneficial agents include doxorubicin, epirubicin, liposomal doxorubicin
ADA	Microarray	Increased (4.05)	Beneficial agents include pentostatin
Estrogen receptor, progesterone receptor, androgen receptor	Negative		Classic hormone therapies are not logical for use with the tumor
** Approval for the research studies on the tumor and on the patient was given by the IRB at MSKCC (New York City, NY), and compliance with HIPAA regulations was met.			

Supplementary Table 1. Summary of findings on the original hFL-HCC cells used to establish the transplantable tumor line. The data on the patients were provided by Memorial Sloan Kettering.

		AHEP H0173	AHEP H301	AHEP H0965	FL-HCC 406	FL-HCC 4628	FL-HCC 469	FL-HCC 470
RNA	RNA Integrity (RIN)	5.6	6.3	8	9.1	7.2	8.9	8.4
	260/280	2.02	2.06	2.03	2.04	2.07	2.04	2.03
	260/230	2.12	1.99	1.83	1.8	2.12	1.71	1.77
Mapping Statistics	By mapping uniqueness:total_reads	208335328	85379820	144734260	194589142	237802258	181373672	231723092
	By mapping uniqueness:unique	189162908	72464257	122993552	161918260	206251240	157412702	157297023
		90.80%	84.87%	84.98%	83.21%	86.73%	86.79%	67.88%
	By mapping uniqueness:multiple	9449534	5433776	7546785	10358559	12089145	9896473	10374915
		4.54%	6.36%	5.21%	5.32%	5.08%	5.46%	4.48%
	By mapping uniqueness:unmapped_reads	9722886	7481787	14193923	22312323	19461873	14064497	64051154
		4.67%	8.76%	9.81%	11.47%	8.18%	7.75%	27.64%
	By pairing types:paired_reads	186142282	69403598	118008204	161911328	205816540	159154812	152905408
		89.35%	81.29%	81.53%	83.21%	86.55%	87.75%	65.99%
	By pairing types:fusion_paired_reads	6540466	4270104	6766364	4230542	5578916	3119782	3873946
		3.14%	5.00%	4.68%	2.17%	2.35%	1.72%	1.67%
	By pairing types:unpaired_reads	5929694	4224331	5765769	6134949	6944929	5034581	10892584
		2.85%	4.95%	3.98%	3.15%	2.92%	2.78%	4.70%
	By alignment types:unspliced	168767663	68520209	109954433	151414156	192071959	147621538	150158494
	By alignment types:spliced	28875945	8825883	19790767	19703881	24764874	18603659	16439889
	By alignment types:fusion	449898	335621	422284	261906	333346	180252	223473
	Splice junctions:total_splice_junction	206370	119923	123615	198590	223072	199747	209990
	Splice junctions:canonical_splice_junction	204992	119339	122841	196562	220874	197945	208080
		99.33%	99.51%	99.37%	98.98%	99.01%	99.10%	99.09%
	Splice junctions:semi_canonical_splice_junction	668	201	257	770	895	768	781
		0.32%	0.17%	0.21%	0.39%	0.40%	0.38%	0.37%
	Splice junctions:non_canonical_splice_junction	710	383	517	1258	1303	1034	1129
		0.34%	0.32%	0.42%	0.63%	0.58%	0.52%	0.54%
	Indels:small_deletions	49454	21339	27087	59663	76019	58928	68741
	Indels:small_insertions	59819	26769	25377	61382	83653	65736	83252
	Fusion junctions:canonical_fusion_junction	783	477	586	704	750	635	694
		11.75%	10.85%	10.39%	11.27%	10.16%	10.59%	10.56%
	Fusion junctions:semi_canonical_fusion_junction	1400	933	1174	1093	1395	1076	1143
		21.01%	21.23%	20.81%	17.50%	18.90%	17.95%	17.39%
	Fusion junctions:non_canonical_fusion_junction	4482	2985	3882	4450	5235	4283	4737
		67.25%	67.92%	68.81%	71.23%	70.93%	71.45%	72.06%
	Filtered fusions:candidate_fusion	143	65	171	161	171	115	134
	Filtered fusions:well_annotated_fusion	0	0	0	0	0	0	0
	Filtered fusions:not_well_annotated_fusion	0	0	0	0	0	0	0
	Filtered fusions:circular_RNAs	0	0	0	0	0	0	0

Supplementary Table 2. Mapping statistics of the hFL-HCC tumor line. This table provides information on the quality of the RNA samples and the quality of the sequencing data for each sample. RIN is an indicator of degradation (with 1 indicating complete degradation and 10 indicating no degradation). Most of the samples had a RIN > 7, which is high quality. 260/280 and 260/230 both measure purity of the RNA. The values of these ratios for most samples are close to 2, which is the standard for high purity RNA samples. We obtained > 180 million reads for each sample and, on average, >85% of these reads were uniquely mapped. A small percentage of reads were mapped to multiple locations or not mapped at all. The rest of the mapping statistics provides detailed information on the proportion of reads that corresponded to unspliced regions, splice junctions, or fusion junctions. Many of the "candidate fusions" are not high-confidence. As described in the manuscript, only one high-confidence recurrent fusion was detected, and this was *DNAJB1-PRKACA*. It was unique to and present in all the hFL-HCCs we analyzed.

MSKCC samples#		Gender	Age	BMI1	SOX9	PDX1	NIS	SHH	HepPar-1
09-2235	7T	F	52	–	–	+	+	++	+
00-36089	T5	F	39	+	+	–	–	++	+
10-4006	ILN1	M	39	+	+	+	+	++	n.d
96-20578	T2/U5	M	48	+	+	+	+	++	+
02-12363	U1	F	19	–	+	+	+	++	n.d
10-7783	2U	M	34	+	+	+	+	++	+
04-8864	12T	F	16	–	–	–	+	++	+
11-15912	5QLN	F	18	–	+	+	–	++	n.d
04-44613	3T	M	22	–	+	+	+	++	n.d
**Total**				4/9	7/9	7/9	7/9	9/9	5/5

Supplementary Table 3. Summary of IHC assays on paraffin sections of original blocks (primary FL-HCCs). The IHC assays on paraffin sections of hFL-HCCs from 9 donors indicated that all are positive for sonic hedgehog (SHH) and, of those assayed, all are positive for HepPar-1. The majority of the tumors (7/9) were positive for SOX9, PDX1, and for NIS, and 4/9 for BMI1. There were two distinct patterns of expression consisting of 1) ones in which most or almost all were positive for a given antigen (e.g. HepPar-1, SHH, NIS and SOX9) but with heterogeneous levels of expression or 2) a pattern in which a percentage of the cells were positive (at least 20%) and the remainder negative (e.g. PDX1 and BMI1).

Age at first diagnosis or age at time of recurrence. ++ = positive in most of the cells but heterogeneous levels of expression; + = heterogeneous expression with a percentage positive (at least 20%) and the rest negative; – = negative in all of the cells; **n.d** = not done

		BTSCs AL703	BTSCs FL693	BTSCs NL692	HBs FL626	HBs FL627	HBs FL634	HpSCs FL552	HpSCs FL614	HpSCs FL651
RNA	RNA Integrity (RIN)	9.6	9.6	7	7.3	7.5	8.5	2.9	8.8	8.7
	260/280	2.03	2.06	2.07	2	2.05	2.04	2.03	2.02	2.06
	260/230	2.08	1.36	1.78	1.86	2.22	1.79	1.94	1.77	2.1
Mapping Statistics	By mapping uniqueness:total_reads	279758114	232834772	148367762	184075024	183509836	187351134	114398386	324149614	284046428
	By mapping uniqueness:unique	260085549	216660542	134284274	161172371	159433679	156178098	101431971	302088828	263062558
		92.97%	93.05%	90.51%	87.56%	86.88%	83.36%	88.67%	93.19%	92.61%
	By mapping uniqueness:multiple	12224169	10139682	10228431	12906630	15063641	22709718	8005037	12661535	12481142
		4.37%	4.35%	6.89%	7.01%	8.21%	12.12%	7.00%	3.91%	4.39%
	By mapping uniqueness:unmapped_reads	7448396	6034548	3855057	9996023	9012516	8463318	4961378	9399251	8502728
		2.66%	2.59%	2.60%	5.43%	4.91%	4.52%	4.34%	2.90%	2.99%
	By pairing types:paired_reads	262038166	218234014	139744064	162418406	163616282	168526164	104061522	300160962	262660682
		93.67%	93.73%	94.19%	88.23%	89.16%	89.95%	90.96%	92.60%	92.47%
	By pairing types:fusion_paired_reads	4604952	4222288	1965190	6034016	5249128	3870662	1348986	7677444	6582152
		1.65%	1.81%	1.32%	3.28%	2.86%	2.07%	1.18%	2.37%	2.32%
	By pairing types:unpaired_reads	5666600	4343922	2803451	5626579	5631910	6490990	4026500	6911957	6300866
		2.03%	1.87%	1.89%	3.06%	3.07%	3.46%	3.52%	2.13%	2.22%
	By alignment types:unspliced	230859927	196594633	132188958	150174648	147218330	152515550	101169883	267044650	237016667
	By alignment types:spliced	40455375	29298084	11604219	23049405	26376725	25591851	7606650	46649200	37560644
	By alignment types:fusion	160510	153538	195096	384120	384891	274785	225009	232682	227604
	Splice junctions:total_splice_junction	263886	235209	190189	186383	170225	197909	114692	255048	236756
	Splice junctions:canonical_splice_junction	262125	233330	188853	185251	169182	196711	113953	253083	234949
		99.33%	99.20%	99.30%	99.39%	99.39%	99.39%	99.36%	99.23%	99.24%
	Splice junctions:semi_canonical_splice_junction	1168	1032	643	531	470	675	242	1186	1048
		0.44%	0.44%	0.34%	0.28%	0.28%	0.34%	0.21%	0.47%	0.44%
	Splice junctions:non_canonical_splice_junction	593	847	693	601	573	523	497	779	759
		0.22%	0.36%	0.36%	0.32%	0.34%	0.26%	0.43%	0.31%	0.32%
	Indels:small_deletions	81300	87496	51082	48922	42371	48601	75307	91080	84679
	Indels:small_insertions	88722	102778	77333	68537	44420	61787	50989	89699	84225
	Fusion junctions:canonical_fusion_junction	693	609	691	625	818	654	1043	977	886
		11.17%	11.18%	9.97%	11.14%	11.51%	12.44%	10.41%	13.63%	12.94%
	Fusion junctions:semi_canonical_fusion_junction	1402	1278	1462	1260	1719	1234	2200	1627	1444
		22.60%	23.46%	21.10%	22.46%	24.19%	23.47%	21.96%	22.70%	21.08%
	Fusion junctions:non_canonical_fusion_junction	4108	3561	4776	3726	4570	3369	6777	4563	4519
		66.23%	65.36%	68.93%	66.41%	64.30%	64.09%	67.63%	63.67%	65.98%
	Filtered fusions:candidate_fusion	203	117	47	94	134	91	51	213	186
	Filtered fusions:well_annotated_fusion	0	0	0	0	0	0	0	0	0
	Filtered fusions:not_well_annotated_fusion	0	0	0	0	0	0	0	0	0
	Filtered fusions:circular_RNAs	0	0	0	0	0	0	0	0	0

Supplementary Table 4. Mapping statistics from the lineage stages of the liver. This table provides information on the quality of the RNA samples and the quality of the sequencing data for each sample. RIN is an indicator of degradation (with 1 indicating complete degradation and 10 indicating no degradation). Most of the samples had a RIN > 7, which is high quality. 260/280 and 260/230 both measure purity of the RNA. The values of these ratios for most samples are close to 2, which is the standard for high purity RNA samples. We obtained > 180 million reads for each sample and, on average, >85% of these reads were uniquely mapped. A small percentage of reads were mapped to multiple locations or not mapped at all. The rest of the mapping statistics provides detailed information on the proportion of reads that corresponded to unspliced regions, splice junctions, or fusion junctions. Many of the "candidate fusions" are not high-confidence. As described in the manuscript, only one high-confidence recurrent fusion was detected, and this was *DNAJB1-PRKACA*. It was unique to the hFL-HCCs.

		TCGA_FLHCC_130620	TCGA_FLHCC_131008	TCGA_FLHCC_140417	FLHCC_Xu_et_al
Mapping Statistics	By mapping uniqueness:total_reads	120389810	159319018	133337286	142056616
	By mapping uniqueness:unique	110762107	146639756	110845423	115237206
		92.00%	92.04%	83.13%	81.12%
	By mapping uniqueness:multiple	5680385	8088181	9159830	4307539
		4.72%	5.08%	6.87%	3.03%
	By mapping uniqueness:unmapped_reads	3947318	4591081	13332033	22511871
		3.28%	2.88%	10.00%	15.85%
	By pairing types:paired_reads	112254218	150785042	116638082	108441200
		93.24%	94.64%	87.48%	76.34%
	By pairing types:fusion_paired_reads	1644836	1048760	821300	991212
		1.37%	0.66%	0.62%	0.70%
	By pairing types:unpaired_reads	2543438	2894135	2545871	10112333
		2.11%	1.82%	1.91%	7.12%
	By alignment types:unspliced	101142388	133078107	109601258	99054955
	By alignment types:spliced	14784005	21114941	9752765	19237781
	By alignment types:fusion	165461	149218	241187	158228
	Splice junctions:total_splice_junction	180491	181433	169699	180905
	Splice junctions:canonical_splice_junction	179683	180508	168850	179901
		99.55%	99.49%	99.50%	99.45%
	Splice junctions:semi_canonical_splice_junction	546	598	405	421
		0.30%	0.33%	0.24%	0.23%
	Splice junctions:non_canonical_splice_junction	262	327	444	583
		0.15%	0.18%	0.26%	0.32%
	Indels:small_deletions	41928	46768	50866	58263
	Indels:small_insertions	53994	52455	53915	45687
	Fusion junctions:canonical_fusion_junction	1216	1355	1453	2687
		11.33%	11.19%	9.64%	38.27%
	Fusion junctions:semi_canonical_fusion_junction	2060	2135	2396	1146
		19.19%	17.63%	15.90%	16.32%
	Fusion junctions:non_canonical_fusion_junction	7458	8622	11224	3189
		69.48%	71.19%	74.46%	45.41%
	Filtered fusions:candidate_fusion	269	502	263	1041
	Filtered fusions:well_annotated_fusion	0	0	0	0
	Filtered fusions:not_well_annotated_fusion	0	0	0	0
	Filtered fusions:circular_RNAs	0	0	0	0

Supplementary Table 5. Mapping statistics of primary hFL-HCCs. This table provides information on the quality of the sequencing data for each primary hFL-HCC. We obtained > 120 million reads for each sample and, on average, >85% of these reads were uniquely mapped. A small percentage of reads were mapped to multiple locations or not mapped at all. The rest of the mapping statistics provides detailed information on the proportion of reads that corresponded to unspliced regions, splice junctions, or fusion junctions. Many of the "candidate fusions" are not high confidence. However, *DNAJB1-PRKACA* was found with high confidence in all 4 primary hFL-HCC samples.

Liver ⟷ Pancreas

**Property**	**hHpSCs (in Canals of Hering)**	**hBTSC Subpopulations (in Peribiliary Glands)**			**Committed Progenitors (in Pancreatic Duct Glands)**	**hFL-HCC Cells**
**Endodermal Markers**	SOX9, LGR5, HNF4A, FOXL1	SOX9, SOX17, LGR5, FOXL1, HNF4A	SOX9, SOX17, PDX1, FOXL1, HNF4A	SOX9 PDX1 LGR5 FOXL1 HNF4A	PDX1, LGR5	SOX9, SOX17, PDX1, LGR5, FOXL1, HNF4A
**Markers of Epithelia**	CK 8 and 18, CK 7 and 19, E-cadherin					
**Cell Adhesion Molecules**	NCAM, EpCAM+, ITGB1 (CD29)	NCAM, EpCAM+	NCAM, EpCAM-	NCAM, EpCAM+	EpCAM+	NCAM, VCAM, EpCAM ± (negligible)
		ITGA6 (CD49f), ITGB4, ITGB1 (CD29)				ITGA6 (CD49f), ITGB4, ITGB1 (CD29)
**Pluri-potency Genes**	OCT4, NANOG, SALL4	KLF4/KLF5, OCT4, NANOG, SALL4, BMI1, TROP-2			None	KLF4/KLF5, OCT4, NANOG, SALL4, BMI1,
**Other Stem Cell Markers**	CXCR4, CD133, Hedgehog proteins (Indian, Sonic), ALDH	CXCR4, CD133, Hedgehog proteins (Indian and Sonic), ALDH			None	CD133, Sonic Hedghog, ALDH
**Protein Matrix Components Receptors**	Laminin, type III collagen, Oncostatin M receptor	Laminin, Oncostatin M receptor Others not yet tested			Fetal islets : collagens IV, V, VI, Laminin, Nidogen, elastin, fetal acinar cells: fibrillar collagens, fibronectin	Laminin, Oncostatin M receptor, CD68
**GAGs/PGs**	Minimally sulfated CS-PGs, Hyaluronans	Hyaluronans, CD44, Others Not yet Tested			Hyaluronans, CD44, fetal islets have syndecans (HS-PG-1 and 3), glypicans,	Hyaluronans, CD44, Syndecan-1 (HS-PG-1)

				fetal acinar cells have CS-PGs	
**Liver-specific Traits**	Albumin +/-, AFP-, HNF4A, HepPar1, KRT7, DLK1	KRT7, HNF4A		None	AFP-, HNF4A, HepPar-1, KRT7
**Pancreatic-specific Traits**	PDX1	PDX1	PDX1, ISL1, NGN3	PDX1, NGN3, MAFA, MUC6, Nkx6, PTF1a, GLUT2	PDX1, KRT20, NGN3
**Sodium-dependent Iodide Transporter (NIS)**	Low levels but evident in all lineage stages of the stem cells.			Weak or none	Strong protein expression but low mRNA levels
**Multidrug Resistance Genes**	MDR-1, ABCG2			None	MDR-1, ABCG2

Supplementary Table 6. Phenotypic profile of normal hepatic and biliary stem/progenitor cells versus human hFL-HCCs. All of the traits given for the hFL-HCCs are as demonstrated in this manuscript. Those for normal subpopulations of hBTSCs, hHpSCs and hHBs are given in part here but also in various prior publications. [1, 2, 3, 4, 5, 6, 8, 28, 29, 30, 31, 32]

HpSCs=Hepatic Stem Cells, BTSCs=Biliary Tree Stem Cells

Name	Clone	Host / isotype	Manufacture	Catalog No.
APC-CD13	WM15	Mouse IgG1	eBioscience	17-0138
PE-CD24	ML5	Mouse IgG2a	BD Biosciences	555428
FITC-CD29 (Integrin β1)	TS2/16	Mouse IgG1	eBioscience	11-0299
APC-CD44	BJ18	Mouse IgG1	BioLegend	338805
FITC-CD49f (Integrin α6)	GoH3	Rat IgG2a	BD Biosciences	555735
FITC-CD54 (ICAM)	HA54	Mouse IgG1	BioLegend	353107
APC-CD56 (NCAM)	MEM-188	Mouse IgG2a	Abcam	ab25358
FITC-CD90 (THY-1)	5E10	Mouse IgG1	eBioscience	11-0909
APC-CD117 (c-KIT)	YB5.B8	Mouse IgG1	BD Biosciences	17-1179
APC-CD133/1	AC133	Mouse IgG1	Miltenyi Biotec	130-090-8
APC-CD184 (CXCR4)	12G5	Mouse IgG2a	eBioscience	17-999
APC-CD324 (E-cadherin)	67A4	Mouse IgG1	Miltenyi Biotec	130-091-2
FITC-CD326 (EpCAM)	VU-1D9	Mouse IgG1	Stem Cell Technologies	10109
PE-TROP-2	77220	Mouse IgG2a	R&D	FAB650P
PE-LGR5	2A2	Mouse IgG1	Origene	TA400001

Supplementary Table 7. Antibodies used for flow cytometric analyses.
APC=allophycocyanin; PE=R-phycoerythrin; FITC=fluorescein isothiocyanate;

Antibody	Species	Isotype	Manufacture	Catalog#	Reactivity	Retrieval
ABCG2	Mouse	IgG2a	Millipore	MAB4146	H	CB
AFP	Mouse	IgG2a	SIGMA	A-8452	H, D,P but not M	CB
BMI1	Rabbit	IgG	Abcam	ab97729	H	CB
CD44	Mouse	IgG2a	Abcam	ab6124	H	CB
CD68	Mouse	IgG3	DAKO	M0876	H	CB
CK7	Mouse	IgG1	DAKO	M7018	H	CB
CK18	Mouse	IgG1	DAKO	M7010	H	CB
CK19	Mouse	IgG2a	Abcam	ab7754	H	CB
E-cadherin	Mouse	IgG2b	Abcam	ab8993	H	CB
EpCAM	Mouse	IgG1	Cell Signaling	#2929	H	CB
HepPar-1	Mouse	IgG1	DAKO	M7158	H	CB
KLF4	Rabbit	IgG	NOVUS	NBP1-83940	H	CB
LGR5	Rabbit	IgG	NOVUS	NBP1-28904	H	CB
MDR-1	Mouse	IgG2a	Abcam	ab10333	H	EDTA
MUC6	Mouse	IgG1	Santa Cruz	sc-33668	H	CB
NANOG	Mouse	IgG1	Cell Signaling	#4893	H	EDTA
NCAM	Mouse	IgG1	DAKO	M7304	H	CB
NGN3	Rabbit	IgG	NOVUS	NBP1-90073	H	EDTA
OCT4	Rabbit	IgG	Cell Signaling	#2750	H	EDTA
PDX1	Rabbit	IgG	NOVUS	NBP1-95578	H	CB
SALL4	Mouse	IgG1	Abcam	ab57577	H	EDTA
SHH	Rabbit	IgG	Millipore	04-971	H	CB
SOX2	Rabbit	IgG	Cell Signaling	#3579	H	CB
SOX9	Rabbit	IgG1	SIGMA	HPA001758	H	CB
SOX17	Mouse	IgG1	Abcam	ab84990	H	CB
SYNDECAN-1 (HS-PG-1)	Goat	IgG	R&D	AF2780	H	CB
VCAM-1	Mouse	IgG1	Santa Cruz	sc-13160	H	CB

Supplementary Table 8. Antibodies used for immunohistochemistry.
H=human, D=dog, M=mouse, P=pig

Name	F/R	Primer Sequence	Product length	GenBank Accession
CD44	F	TGCCGCTTTGCAGGTGTAT	66	NM_000610.3
	R	GGCCTCCGTCCGAGAGA		
CDH1	F	TCACAGTCACTGACACCAACGA	67	NM_004360
	R	GGCACCTGACCCTTGTACGT		
CFTR	F	AAAAGGCCAGCGTTGTCTCC	170	NM_000492.3
	R	TGAAGCCAGCTCTCTATCCCA		
KRT7	F	TGCTGCCTACATGAGCAAGGT	99	NM_005556.3
	R	TCTGTCAACTCCGTCTCATTGAG		
KRT18	F	GCCCGCTACGCCCTACA	57	NM_000224.2
	R	TGACTCAAGGTGCAGCAGGAT		
KRT19	F	CCGCGACTACAGCCACTACT	97	NM_002276.4
	R	GTCGATCTGCAGGACAATCC		
LGR5	F	GAGGATCTGGTGAGCCTGAGAA	151	NM_001277227.1
	R	CATAAGTGATGCTGGAGCTGGTAA		
NANOG	F	AAATCTAAGAGGTGGCAGAAAAACA	60	NM_024865.2
	R	CTTCTGCGTCACACCATTGC		
PDX1	F	CCCATGGATGAAGTCTACC	262	NM_000209.3
	R	GTCCTCCTCCTTTTTCCAC		
POU5F1	F	GAGAGGCAACCTGGAGAATTTG	58	NM_001173531.1
	R	GATCTGCTGCAGTGTGGGTTT		
PROM1	F	TCCACAGAAATTTACCTACATTGG	77	NM_001145851.1
	R	CAGCAGAGAGCAGATGACCA		
SOX2	F	AAATGGGAGGGGTGCAAAAGAGGAG	112	NM_003106.3
	R	CAGCTGTCATTTGCTGTGGGTGATG		
TACSTD1	F	GACTTTTGCCGCAGCTCAGGAAG	135	NM_002354.1
	R	GCCAGCTTTGAGCAAATGACAGTATTTTG		
GAPDH	F	AAGGTGAAGGTCGGAGTCAA	108	NM_002046.3
	R	AATGAAGGGGTCATTGATGG		

Supplementary Table 9. Primers used for qRT-PCR.
F=forward; R=reverse

Supplementary Note 1.

Original hFL-HCC used for the transplantable tumor line

A male patient, age 25, presented in August, 2008 to Greenwich Hospital (Yale/New Haven Hospital, Greenwich, CT) with acute swelling of his right lower leg. During the initial evaluations, he was found to have an extensive venous thrombus extending from his right ankle into his inferior vena cava. A CT of his chest/abdomen/pelvis revealed multiple small pulmonary emboli and a large mass in his left liver with evidence of metastatic disease in the peri-hepatic lymph nodes, omentum and peritoneum. He had a venous filter inserted above the thrombus, was started on anticoagulation and transferred to Memorial Sloan Kettering Cancer Center (MSKCC, New York City, NY) for further studies and therapy. The patient underwent a liver biopsy resulting in a pathologic diagnosis of hepatocellular carcinoma (HCC) and subsequently had an extensive debulking procedure which included a left hepatic lobectomy and debulking of periotoneal and omental nodules. Macroscopically, the peritoneal and omentum nodules proved to be tumors, and histologically revealed tumor tissue consistent with a diagnosis of FL-HCC. Surgical pathology revealed tumor cells positive for HepPar1 and cytokeratin 7 (CK7). Analyses of EMA (epithelial membrane antigen) and AFP (α-fetoprotein) were not conclusive and neither were tissues stained for reticulin, iron, or PAS-3. A summary of the characterization of the original tumor by the pathologists at MSKCC is given in **Supplementary Table 1**. A later round of biopsies resulted in similar pathology reports. These included cytology on pleural fluid found replete with tumor cells; these also had a pathology consistent with a diagnosis of FL-HCC.

The patient was subsequently treated with various oncoloytic agents including sorafenib, doxorubicin, gemcitabine, cisplatinum, 5-FU, bevacizumab, and thalidomide with limited or no responses. In September of 2009 after showing progressive enlargement in the peri-hepatic and retroperitoneal nodes, recurrent disease in the liver, and increasing size of omental and peritoneal nodules, the patient returned to MSKCC to obtain further tissue for tumor sensitivity studies and debulking. Biopsies were taken, but his disease was too extensive for debulking. Paclitaxel and thalidomide were then started based on sensitivity studies but were poorly tolerated with continued disease progression, so treatment was stopped. After 4 months it was realized that he had widely disseminated disease especially in the ascites fluid. In early February, 2010, a palliative

paracentesis was done for massive ascites, and approximately 5 liters of fluid were removed and transferred to several researchers, including those in the UNC research lab, in hopes that studies on the tumor might identify alternate treatments. A week later the patient passed away peacefully.

Supplementary Note 2.

Background on normal human biliary tree stem cells

More detailed studies and reviews on biliary tree stem cells (hBTSCs) are given elsewhere[1, 2, 3, 4, 5, 6, 7, 8, 9, 10, 11, 12, 13, 14]. In brief, the biliary tree contains stem cell niches, peribiliary glands (PBGs), mucinous glands scattered as ***intramural PBGs*** within the walls of the bile ducts and also found as ***extramural PBGs*** tethered to the surface of the bile ducts[15, 16, 17]. The phenotypes of the cells within the intramural PBGs can be relatively homogeneous in some sites (e.g. hepato-pancreatic common duct and intrahepatic, large bile ducts) and quite heterogeneous in other sites (e.g. cystic duct, common duct, hepatic duct)[6, 7, 9]. The pattern of phenotypic traits of the PBG cells has been found to indicate maturational lineages in a **radial axis** from the fibromuscular layer within the duct walls to the lumen of the bile ducts and in a **proximal (duodenum)-to-distal axis** from the duodenum to either liver or pancreas[1, 4]

The PBGs deepest within the bile ducts and near the fibromuscular layer contain the most primitive hBTSCs, those that co-express transcription factors for both liver and pancreas (e.g. SOX17, PDX1), that also co-express multiple pluripotency genes (e.g. OCT4, SOX 2, KLF4/KLF5, NANOG)[1, 2, 4] and that have surface markers of CD44, the hyaluronan receptor, and sodium iodide symporter (NIS). These cells do not express epithelial cell adhesion molecule (EpCAM) or even LGR5. We refer to these as primitive hBTSCs or, alternatively, stage 1 hBTSCs. They transition to cells expressing LGR5, CD44 and NIS (stage 2 hBTSCs) and then to ones positive for also for EpCAM (stage 3 hBTSCs). These are found at levels that are intermediate within the bile ducts. With transition to the bile duct lumens, there is acquisition of cells with mature phenotypic markers. If the ducts are near or within the liver, the mature markers are those for liver; if they are within the hepato-pancreatic common duct, the mature markers are pancreatic.

As noted in the main text, all cultures of hBTSCs comprise cells that express all of the biomarkers noted for stage 2 or for stage 3 hBTSCs. No colonies have been identified that are devoid of LGR5 or EpCAM.

The reason for the absence of stage 1 hBTSCs in the cultures is unknown but is hypothesized to be either that distinct culture conditions are required for them or that culturing (or proliferative state?) of the cells triggers expression of LGR5. Parallel findings have been shown in intestinal stem cell subpopulations in which LGR5+ with and without EpCAM+ expression identify important subpopulations driven by wnt signaling pathways[18]. These are questions being addressed in ongoing studies.

Supplementary Note 3.

Relevance of stem/progenitor cells to liver regeneration

Liver regeneration postnatally has long been known to involve adult parenchymal cells in either hyperplastic (complete cell division) or hypertrophic responses (DNA synthesis with absence of cytokinesis leading to polyploidy) [19]. Hyperplastic responses dominate following injuries to zone 3 parenchymal cells (and even more so if zone 2 cells are also damaged). By contrast, partial hepatectomy elicits a wave of DNA synthesis across liver plates but with minimal, if any, cytokinesis in zones 2 and/or 3 parenchymal cells and so leads to polyploidy that secondarily triggers increased rates of apoptosis [20].

The extent of liver mass is maintained primarily by adult cells in normal turnover (quiescent livers) and following mild injuries, regulated by effects of the Hippo/Yap signaling pathway [21] and by feedback loop signals produced by terminally differentiated hepatocytes, released into bile, carried in the opposite direction from blood flow, and influencing mechanisms periportally. Feedback loop signals identified to date include factors from bile acid metabolism [22] and mechanical effects of bile flow on primary cilia-positive cells [23]. The primary cilia have a base containing hedgehog proteins functionally connected to the Wnt/beta catenin signaling pathway [23]. Mechanical flexing of cilia by bile flow results in inhibition of cell division; reduction or loss of bile flow, as occurs in liver failure, disinhibits the cells and triggers cell division.

A novel marking method has been established by Kaneko et al [24] and enables visualization of unique architectural adaptations of the biliary tree macroscopically and microscopically in response to distinct injuries. The architectural adaptations are many, but representative examples include ones with periportal damage resulting in greater arborization of ducts, whereas damage pericentrally results in extension and lengthening of the ducts. This suggests a multi-stage process in which injuries trigger regenerative responses in the network

of biliary tree stem/progenitor subpopulations and secondarily in their parenchymal cell descendants. The implications are that liver regeneration derives from both stem/progenitors and adult cells. Stem/progenitors can have a role even in normal turnover in quiescent livers or those with mild injuries, since the number of divisions required is small and easily missed [25]. With increasing severity of the injuries, the contributions by stem/progenitor populations are presumed to increase to provide sufficient functional liver tissue for the host to survive [8, 19, 26].

Supplementary References

1. ***Cardinale V*, *et al.* Multipotent stem cells in the extrahepatic biliary tree give rise to hepatocytes, bile ducts and pancreatic islets. *Hepatology* **54**, 2159-2172 (2011).

2. *Carpino G*, et al.* Biliary tree stem/progenitor cells in glands of extrahepatic and intraheptic bile ducts: an anatomical *in situ* study yielding evidence of maturational lineages. *Journal of Anatomy* **220**, 186-199 (2012).

3. Semeraro R*, et al.* Multipotent Stem/Progenitor Cells in the Human Foetal Biliary Tree. *Journal of Hepatology* **220**, 186-199 (2012).

4. Wang Y*, et al.* Biliary Tree Stem Cells, Precursors to Pancreatic Committed Progenitors: Evidence for Life-long Pancreatic Organogenesis. . *Stem Cells* **31**, 1966-1979 (2013).

5. Carpino G*, et al.* Human gallbladder contains multipotent stem/progenitor cells. *Journal of Hepatology* **60**, 1194-2020 (2014).

6. Cardinale V*, et al.* The Biliary Tree: a Reservoir of Multipotent Stem Cells. *Nature Reviews-Gastroenterology and Hepatology* **9**, 231-240 (2012).

7. Furth ME*, et al.* Stem Cell Populations Giving Rise to Liver, Biliary Tree and Pancreas. In: *The Stem Cells Handbook, 2nd Edition* (ed^(eds Sell S). Springer Science Publishers, NY, NY (2013).

8. Turner R*, et al.* Hepatic stem cells and maturational liver lineage biology. *Hepatology* **53**, 1035-1045 (2011).

9. Lanzoni G*, et al.* Clinical Programs of Stem Cell Therapies for Liver and Pancreas. *Stem Cells* **31**, 2047-2060 (2013).

10. Desmet V, Roskams T, Van Eyken P. Pathology of the biliary tree in cholestasis: ductular reactions. In: *Cholestatic liver diseases* (ed^(eds Manns MP, BOyer JL, Jansen PLM, Reichen J). Kluwer Academic Publishers (1998).

11. Roskams TA*, et al.* Nomenclature of the Finer Branches of the biliary Tree: Canals, Ductules, and Ductular Reactions in Human Livers. *Hepatology* **39**, 1739-1745 (2004).

12. Mancinelli R*, et al.* After Damage of Large Bile Ducts by Gamma-Aminobutyric Acid, Small Ducts Replenish the Biliary Tree by Amplification of Calcium-Dependent Signaling and de Novo Acquisition of Large Cholangiocyte Phenotypes. *American Journal of Pathology*, **176**, 1790-800 (2010).

13. Nakanuma Y, Sasaki M. Expression of blood group-related antigens in the intrahepatic biliary tree and hepatocytes in in normal livers and various hepatobiliary diseases. *Hepatology* **10**, 174-178 (1989).

14. Crawford JM. Development of the intrahepatic biliary tree. *Seminars In Liver Disease* **22**, 213-226 (2002).

15. Nakanuma Y, Hoso M, T. S, Sasak IM. Microstructure and development of the normal and pathologic biliary tract in humans, including blood supply. A Review. *Microscope Research Technique* **15**, 552-570 (1997).

16. Terada T, Nakanuma Y, Ohta G. Glandular elements around the intrahepatic bile ducts in man; their morphology and distribution in normal livers. . *Liver* **7**, 1-8 (1987).

17. Terada T, Nakanuma Y. Development of human intrahepatic peribiliary glands. Histological, keratin immunohistochemical, and mucus histochemical analyses. *Laboratory Investigations* **68**, 261-269 (1993).

18. Kemper K, Prasetyanti PR, De Lau W, Rodermond H, Clevers H, Medema JP. Monoclonal antibodies against Lgr5 identify human colorectal cancer stem cells. *Stem cells (Dayton, Ohio)* **30**, 2378-2386 (2012).

19. Miyajima A, Tanaka M, Itoh T. Stem/progenitor cells in liver development, homeostasis, regeneration, and reprogramming. *Cell Stem Cell* **14**, 561-574 (2014).

20. Gupta S. Hepatic polyploidy and liver growth control. *Seminars in Cancer Biology* **10**, 161-171 (2000).

21. Johnson R, Halder G. The two faces of Hippo: targeting the Hippo pathway for regenerative medicine and cancer treatment. *Nature Reviews-Drug Discovery* **13**, 63-79 (2014).

22. Fan M, Wang X, Xu G, Yan Q, Huang W. Bile acid signaling and liver regeneration. *Biochimical and Biophysical Acta* **1849**, 196-200 (2015).

23. Grzelak CA, *et al.* The intrahepatic signalling niche of hedgehog is defined by primary cilia-positive cells during chronic liver injury *Journal of Hepatology* **60**, 143-151 (2014).

24. Kaneko K, Kamimoto K, Miyajima A, Itoh T. Adaptive Remodeling of the Biliary Architecture Underlies Liver Homeostasis *Hepatology* **61**, 2056-2066 (2015).

25. Stueck AE, Wanless IR. Hepatocyte buds derived from progenitor cells repopulate regions of parenchymal extinction in human cirrhosis *Hepatology* **61**, 1696-1707 (2015).

26. Choi TY, Ninov N, Stainier DY, Shin D. Extensive conversion of hepatic biliary epithelial cells to hepatocytes after near total loss of hepatocytes in zebrafish. *Gastroenterology* **146**, 776-788 (2014).

27. Xu L, *et al.* Genomic analysis of fibrolamellar hepatocellular carcinoma. *Human Molecular Genetics* **24**, 50-63 (2015).

28. *****Schmelzer E*, et al.* Human hepatic stem cells from fetal and postnatal donors. *Journal of Experimental Medicine* **204**, 1973-1987 (2007).

29. Schmelzer E, Reid LM. Telomerase activity in human hepatic stem cells , hepatoblasts and hepatocytes from neonatal, pediatric, adult and geriatric donors. *European Journal of Hepatology and Gastroenterology* **21**, 1191-1198 (2009).

30. Schmelzer E, Wauthier E, Reid LM. Phenotypes of pluripotent human hepatic progenitors. *Stem Cell* **24**, 1852-1858 (2006).

31. Turner WS, Schmelzer E, McClelland R, Wauthier E, Chen W, Reid LM. Human hepatoblast phenotype maintained by hyaluronan hydrogels. *Journal of Biomedical Materials* **82**, 156-168 (2007).

32. Sicklick JK*, et al.* Hedgehog signaling maintains resident hepatic progenitors throughout life *American Journal of PhysiologyGastrointestinal Liver Physiology* **290**, G859-G870 (2006).
